# Durum wheat nuclear factor Y (NF-Y) a subfamily: structure, phylogeny, and expression analysis in response to hormones and abiotic stresses

**DOI:** 10.1007/s10142-025-01607-z

**Published:** 2025-05-14

**Authors:** Yosra Chouaibi, Mohamed Taieb Bouteraa, Walid Ben Romdhane, Narjes Baazaoui, Mohammad Y. Alfaifi, Miroslava Kačániová, Natália Čmiková, Anis Ben Hsouna, Stefania Garzoli, Alina Wiszniewska, Rania Ben Saad

**Affiliations:** 1 Centre of Biotechnology of Sfax, Biotechnology and Plant Improvement Laboratory, University of Sfax, B.P ‘1177’, 3018 Sfax, Tunisia; 2 Faculty of Sciences of Bizerte UR13ES47, University of Carthage, BP W, 7021 Jarzouna, Bizerte, Tunisia; 3 Plant Production Department, College of Food and Agriculture Sciences, King Saud University, P.O. Box 2460, 11451 Riyadh, Saudi Arabia; 4 Biology Department, College of Sciences and Arts Muhayil Assir, King Khalid University, 61421 Abha, Saudi Arabia; 5 Biology Department, Faculty of Science, King Khalid University, 61421 Abha, Saudi Arabia; 6 Institute of Horticulture, Faculty of Horticulture, Slovak University of Agriculture, Tr. A. Hlinku 2, 949 76 Nitra, Slovakia; 7 School of Medical & Health Sciences, University of Economics and Human Sciences in Warsaw, Okopowa 59, 01043 Warsaw, Poland; 8 Department of Environmental Sciences and Nutrition, Higher Institute of Applied Sciences and Technology of Mahdia, University of Monastir, 5100 Mahdia, Tunisia; 9 Department of Chemistry and Technologies of Drug, Sapienza University, P.Le Aldo Moro 5, 00185 Rome, Italy; 10 Department of Botany, Physiology and Plant Protection, Faculty of Biotechnology and Horticulture, University of Agriculture in Kraków, Al. Mickiewicza 21, 31-120 Cracow, Poland

**Keywords:** *NF-YA* transcription factor, *TtNF-YAs* gene family, *Triticum turgidum*, Abiotic stress tolerance, Yeast

## Abstract

Nuclear factor-Y (NF-Y) transcription factors are heterotrimeric complexes that are widely distributed in eukaryotes and play essential roles in many biological processes. Although NF-YA proteins have been characterized in numerous plants, their contribution to the response of durum wheat (*Triticum turgidum* ssp. *durum*) to environmental factors has not been reported. Thus, this study was aimed at identification and characterization of *Triticum turgidum TtNF-YA* family members through genome-wide analysis. Twelve *NF-YA* genes were discovered in *Triticum turgidum*. Discovered genes were distributed across eight chromosomes, while their encoded proteins were localized in cell nucleus. Structure and motif pattern analyses revealed that the *TtNF-YA* genes were relatively conserved. The expression of *TtNF-YAs* genes was significantly induced by several stressors and their expression profiles differed in various tissues and at various development stages. Notably, *TtNF-YA2 A-1* and *TtNF-YA2B-1* exhibited the greatest increase in response to Polyethylene glycol, while *TtNF-YA4 A* and *TtNF-YA4B-1* showed the highest increase under salt stress. Additionally, *TtNF-YA5B-1* and *TtNF-YA6 A-1* displayed pronounced upregulation when exposed to exogenous Abscisic acid, suggesting that *TtNF-YA* are involved in a series of cellular and developmental events. This finding was corroborated by the recognition of several *cis*-regulatory elements in the *TtNF-YAs* promoter region, associated with the applied treatments. Overexpression of *TtNF-YA2 A-1*, *TtNF-YA2B-1*, *TtNF-YA4 A*, *TtNF-YA4 A-1, TtNF-YA4B-1*, and *TtNF-YA5 A-2* genes in *Saccharomyces cerevisiae* showed that these genes increase cell tolerance to multiple stresses. Our results will facilitate subsequent functional analysis of *TtNF-YAs* genes, which emerge as promising targets for genetic engineering for increasing wheat tolerance to multiple stresses.

**Supplementary Information:**

The online version contains supplementary material available at 10.1007/s10142-025-01607-z.

## Introduction

Climatic changes and environmental pollution are among the major challenges for crop breeding for future agriculture. To counteract stresses caused by exposure to suboptimal factors, plants have developed a wide range of regulatory mechanisms. Nuclear factor-Y (NF-Y), also known as heme-activating protein (HAP) or CCAAT-binding factor (CBF), is a ubiquitous transcription factor (TF) in eukaryotes. Its expression in plants is significantly modulated in response to abiotic stress, making *NF-Y* genes and their products key regulators of defense processes (Wu et al. 2018; Hwang et al. 2019). The plant NF-Y complex consists of three subunits, NF-YA, NF-YB, and NF-YC, which are linked to the CCAAT box of the promoter region of specific genes. The combinations of the subunits, resulting from mutual interactions, regulate the expression of multiple genes (Nardini et al. 2013). In contrast to animals and yeasts, where each subunit is encoded by a single gene, in plants, several genes were found to encode each subunit (Laloum et al. 2013). The first report on plant NF-Y identification was on *Brassica napus,* where 33 *NF-Ys* genes were described (14 *BnNF-YAs*, 14 *BnNF-YBs*, and 5 *BnNF-YCs*) (Albani and Robert 1995). Since then, genes belonging to this family have been identified in other plant species, and it became evident that the particular number of NF-Ys substantially varies among them (Siefers et al. 2009; Cao et al. 2011; Yang et al. 2017a; Liang et al. 2014; Quach et al. 2015). Analysis of gene structure allowed classification the genes into respective subfamilies (A, B, and C). The sequences of the A subunits are generally longer than those of the remaining subfamilies (Nardone et al. 2017). The *NF-YA* has been shown to be involved in numerous aspects of plant growth and development, including seed formation, root progression and flowering, as well as response to environmental conditions (Zhou et al. 2020). Regulatory action of *NF-YA* influences phytohormonal signaling and the level of antioxidant activity, as well as the expression of wide range of stress-related genes (Zhang et al. 2023). In *Arabidopsis thaliana*, the *AtNF-YA1*, *AtNF-YA5*, *AtNF-YA6*, and *AtNF-YA9* regulate seed growth and germination (Mu et al. 2013), while the *AtNF-YA2* and *AtNF-YA10* genes affect leaf growth through the activation of auxin signaling (Zhang et al. 2017). *AtNF-YA2*, *AtNF-YA3* and *AtNF-YA5* are also involved in nitrogen nutrition (Laloum et al. 2013). In addition, *NF-YA* genes are recognized as stimulators of flowering (Siriwardana et al. 2016; Su et al. 2018). The NF-YA family appears to be an important class of genes particularly implicated in response to water deficit and osmotic disturbances (Alam et al. 2015). Expression of wheat *TaNF-YB3* and rice *OsNF-YA7* affected the ABA-associated signaling pathway, resulting in increased tolerance to drought (Lee et al. 2015; Yang et al. 2017b). Ni et al. (2013) showed that the soybean *GmNF-YA3* transgene positively regulated drought tolerance stress in *Arabidopsis thaliana* (Ni et al. 2013). Additionally, the *ZmNF-YA1* subunit controlled the expression of several stress-related genes to improve water stress tolerance (Wang et al. 2018). Other studies revealed that in *Triticum aestivum*, the expression of some *TaNF-Y* genes is organ-specific, or is induced only in response to drought, while other genes are ubiquitously expressed (Stephenson et al. 2007). Zhao et al. (2022) demonstrated that the *TaNF-YA7* gene was expressed in response to water stress, contributing to controlled stomatal orientation by reducing water loss, and maintaining ROS balance. Furthermore, according to Ma et al. (2015a), overexpression of the *TaNF-YA10* gene in *A. thaliana* enhanced overall tolerance to abiotic stress. In tobacco, the *CsNF-YA5* gene showed antioxidant activity, which was explained by a reduction in H_2_O_2_ content under water stress conditions (Pereira et al. 2018). In *Ginkgo biloba*, *GbNF-YA* genes have been shown to be expressed in response to salt, water, and heat stress (Wang et al. 2023). Also, maize *ZmNF-YA12* gene has been found to be involved in salt and water stress responses by stimulating the expression of stress-related genes (Zhang et al. 2022). 

Given the importance of *NF-YA* in plant biology, the identification and characterization of the *NF-YA* gene family in the durum wheat genome become desirable for further improvement of this essential crop. To our knowledge, little is known about *NF-YA* transcription factors and their homologs in *T. turgidum*, despite the availability of its genome (12 Gb). Also, the role of NF-YA proteins in the response to several constraints remains elusive in this species. In this study, at first, we analyzed the gene structure, motif composition, conserved domains, chromosomal location, subcellular localization, proteins interaction network, phylogenetic relationships, and *cis*-regulatory elements in the promoter regions of the different *TtNF-YA* subfamilies in *T. turgidum*. Secondly, we evaluated the responsiveness of 12 *TtNF-YA* genes to abiotic stress and phytohormone treatments, and their expression in various tissues. Based on these expression profiles, we screened six *TtNF-YA* genes that responded strongly to abiotic stress (salt, osmotic, heat, and cold) for their ability to improve stress tolerance in a heterologous eukaryotic system (*S. cerevisiae*). Our goal was to boost the understanding on the genome-wide evolution of NF-YA family members and *TtNF-YA* genes expression analysis in *T. turgidum* under stress conditions, which is an important step for further investigation of their functions.

## Materials and methods

### Plant material, stress treatments and growth conditions

Tunisian durum wheat seeds cultivar “Karim” were surface sterilized and germinated on wet Whatman paper pieces in Petri dishes as described by Bouteraa et al. (2023). To understand how the twelve *TtNF-YA* genes respond to stress, ten-day-old wheat seedlings grown in nutrient solution using a hydroponic system were exposed to various stressors, including salinity, osmotic, cold, heat, abscisic acid (ABA) and methyl jasmonate (MejA). The salt (150 mM NaCl) and osmotic stress (20% (w/v) PEG 6000) were applied as described by Ben Romdhane et al. (2022). For cold and heat treatments, seedlings were incubated at 4 °C or at 37 °C. The seedlings were grown in a controlled environment chamber (phytotron) under the following conditions: temperature (25 ± 5 °C), light intensity (280 mmol. m^−2^. s^−1^), photoperiod (16 h light/8 h dark), and relative humidity (60 ± 10%). Leaves of the plants were sprayed with the phytohormones ABA (100 µM) and MejA (100 µM). A separate group of plants served as the control and received only water spray. All test plants were harvested at designated time points following stress treatment: 3, 12, and 24 h. Therefore, to analyze tissue-specific expression, various plant parts were collected from greenhouse-grown individuals: leaves, stems, roots, spikes, anthers, and even developing seeds at 21 days after flowering (anthesis). Each tissue type was collected separately. To preserve RNA integrity, samples were immediately frozen in liquid nitrogen and stored at −80 °C.

### Identification of TtNF-YA proteins in durum wheat and chromosomal location

Durum wheat TtNF-YA family members were discovered by mining the Ensembl Plants database (https://plants.ensembl.org/index.html). Protein sequences for TaNF-YA proteins, previously identified by Stephenson et al. (2007), available in the Grain Genes database (https://wheat.pw.usda.gov/), served as a search query against the TtNF-YA proteins using the BLAST tool. A stringent E-value threshold of E^−50^ was employed to ensure high-quality matches. The protein sequences of TtNF-YA subunits were analyzed using InterPro (https://www.ebi.ac.uk/interpro/) and SMART (http://smart.embl-heidelberg.de/) to confirm the presence of the PF02045 NF-YA domains. The chromosomal locations of each *TtNF-YA* gene were plotted using PhenoGram Plot server by determining the length of all chromosomes and the position of each gene. For a more comprehensive view, a bioinformatics workflow outlining the identification, in silico analysis, and expression analysis of *TtNF-YA* genes is reported in Fig. S1.

### Phylogenetic analysis and motif conservation of TtNF-YA family members

To construct a phylogenetic tree encompassing the evolutionary relationships of TtNF-YA proteins, protein sequences from NF-YA proteins in wheat (*Triticum aestivum*), *Arabidopsis thaliana*, rice (*Oryza sativa*), sorghum (*Sorghum bicolor*), and barley (*Hordeum vulgare*) were retrieved alongside the TtNF-YA sequences (Table S2). The maximum likelihood method implemented within MEGA11 software was employed for tree construction, incorporating 1000 bootstrap replicates to enhance the reliability of the branching patterns (Tamura et al. 2021). In addition, the ClustalW algorithm was used to align the sequences with MEGA11. Data provided with bootstrap values were presented using the Interactive Tree of Life (ITOL) server (https://itol.embl.de/itol_account.cgi). For comparative synteny analysis, sequence similarity was predicted using the Circoletto server (https://bat.infspire.org/circoletto/). TtNF-YA motif preservation was performed using the MEME v5.4.1 tool (Bailey et al. 2015).

### Characterization of TtNF-YA family members and their in-silico network interactions

To understand the biophysical characteristics of the TtNF-YA proteins, several key parameters were determined by the ExPASy bioinformatics web tool. The physical properties assessed were molecular weight (MW) and instability index (II). On the chemical side, the isoelectric point (pI) and the grand average of hydropathicity (GRAVY) were analyzed (Artimo et al. 2012). In addition, the subcellular location of each TtNF-YA protein was estimated by the subcellular localization predictor BUSCA server (https://busca.biocomp.unibo.it/) (Savojardo et al. 2018). The Swiss model server was used to predict the three-dimensional (3D) structure of TtNF-YA proteins (https://swissmodel.expasy.org/). The *in-silico* protein–protein interactions were determined by the STRING (Search Tool for the Retrieval of Interacting Genes/Proteins) database (https://string-db.org/) based on the registered experimental data using the 12 TtNF-YA protein sequences as queries to find their interactors in the *Triticum aestivum* proteome.

### Gene organization and promoter region

Homologs, paralogs, and orthologs of each *TtNF-YA* gene were identified using the Plant Compara tool within Ensembl Plants. The evolutionary pressure acting on these genes was then analyzed with the TBtools v1.095 software to calculate evolutionary pressure (Chen et al. 2020). Furthermore, the exon–intron structures of the *TtNF-YA* genes were examined using the online resource GSDS2.0 (http://gsds.gao-lab.org) (Hu et al. 2015). To identify potential regulatory elements influencing *TtNF-YA* gene expression, promoter sequences were extracted from Ensembl Plants for a region of 2000 bp upstream of each gene's start codon. These sequences were then analyzed using two online databases: PlantCare (https://pubmed.ncbi.nlm.nih.gov/9847207/) (Lescot et al. 2002) and PLACE (https://www.ncbi.nlm.nih.gov/pmc/articles/PMC8133646/) (Higo et al. 1999). The number of putative cis-regulatory elements discovered within each *TtNF-YA* gene promoter was subsequently visualized using TBtools software (Chen et al. 2020).

### Transcriptomic analysis of TtNF-YAs genes

Plant RNA was extracted using TRIzol (Invitrogen). The RNA was treated with DNase I (MBI Fermentas, USA) at 37 °C for 15 min to remove any remaining genomic DNA. Two micrograms of total RNA were then reverse-transcribed into cDNA using M-MLV reverse transcriptase (Thermo Fisher Scientific). The resulting cDNA was diluted 1:5 and amplified with gene-specific primers designed using Primer 3 (http://primer3plus.com/cgi-bin/dev/primer3plus.cgi) and SYBR Green RT-PCR master mix (Roche). The LightCycler 480 real-time PCR system (Roche) was employed for all quantitative RT-qPCR assays, which were performed in triplicate following the methodology established by Ben Saad et al. (2019). The PCR cycling conditions were as follows: 95 °C for 3 min, followed by 40 cycles of 95 °C for 20 s, 60 °C for 30 s, and 72 °C for 1 min. A melting curve was routinely performed after 40 cycles to verify primer specificity. The relative expression levels of the twelve *TtNF-YAs* were calculated by using the 2^−ΔΔ*C*T^ method, where CT indicates the cycle threshold, described by Livak and Schmittgen (2001) and normalized using the cell division control protein (AAA-superfamily of ATPases) (*CDC*, Ta54227) (Giménez et al. 2011). Relative expression ratios are reported from three independent experiments (with three biological repetitions).

### Overexpression of TtNF-YAs genes in yeast

The full-length cDNAs of six *TtNF-YA* genes (*TtNF-YA2 A-1*, *TtNF-YA2B-1*, *TtNF-YA4 A*, *TtNF-YA4 A-1*, *TtNF-YA4B-1*, and *TtNF-YA5 A-2*) were introduced into the pYES2 expression vector (Invitrogen) via the restriction enzymes *EcoR*I/*Xba*I (Table S1) downstream of the GAL1 promoter (inducible by galactose) and used to transform the W303 strain of *S. cerevisiae*, while the control had an empty pYES2 vector. To mobilize the plasmids, the standard PEG lithium acetate method was used (Soni et al. 1993). After transformation, the W303 yeast strains were evaluated for growth using a specific growth medium containing 2% YNB, uracil, and galactose. To assess stress tolerance, colonies confirmed positive through PCR verification were grown overnight in defined media lacking uracil (YNBUra-) until reaching the mid-exponential growth phase. These cultures were then adjusted to an “OD600” of 1 and subjected to serial dilutions using fresh media (10^–2^, 10^–4^, or 10^–6^). Subsequently, 5 µL aliquots from each dilution were plated onto solid YNBUra-Gal medium (drop test). These plates were then supplemented with either 2 M NaCl or 2 M mannitol to induce stress at 30 °C for 4 days, or heat and cold stress (37 °C and 4 °C, respectively, for 2 days). The growth rate on solid media was assessed visually.

## Results

### Identification of TtNF-YA gene members in durum wheat and chromosomal location

**Fig. 1:**
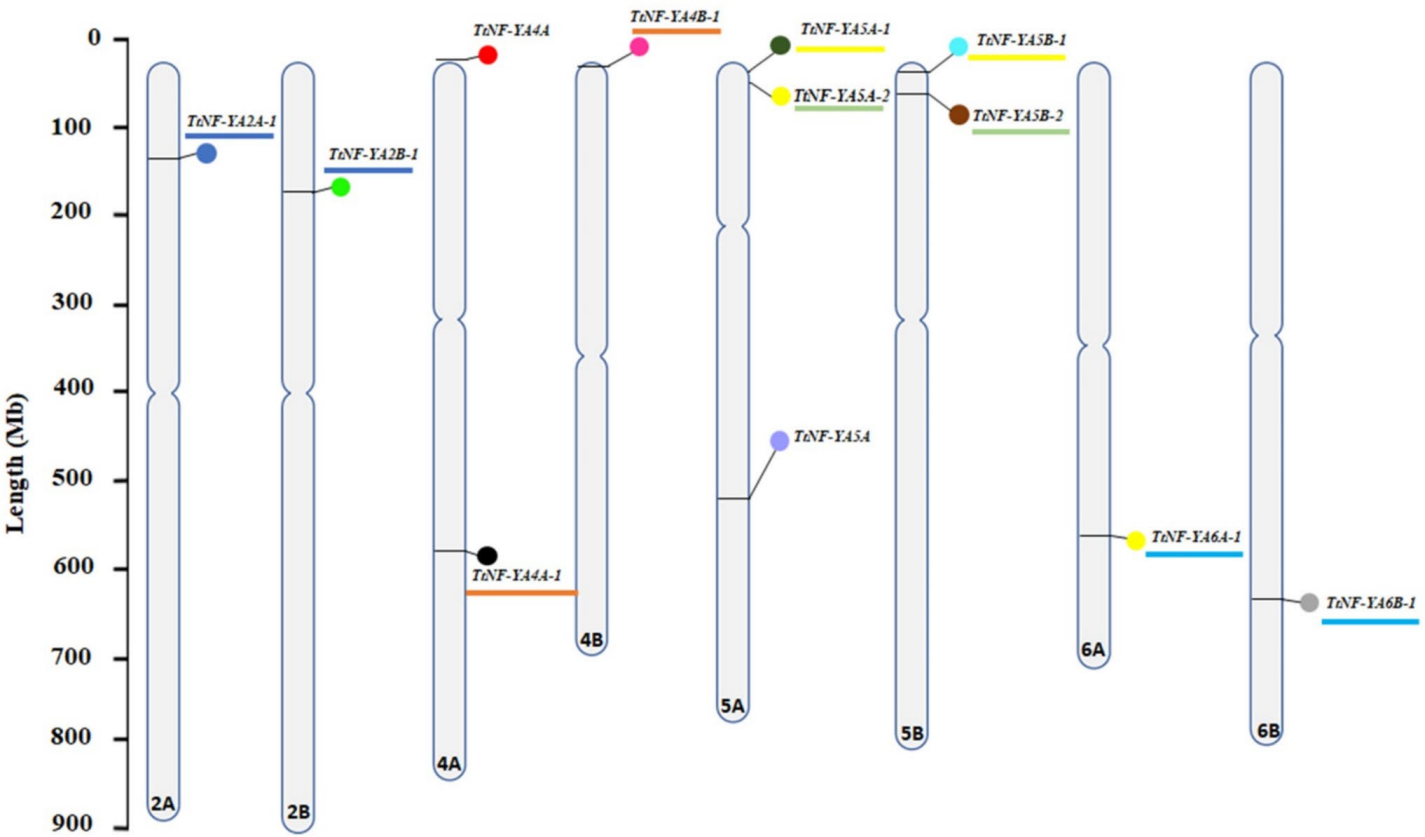
Positions of *TtNF-YA* genes on durum wheat chromosomes. The chromosome scale is displayed on the left in units of millions of base pairs (Mb). Duplicated gene pairs are highlighted with matching colors

Twelve genes were identified and named *TtNF-YA2 A-1, TtNF-YA2B-1, TtNF-YA4 A, TtNF-YA4 A-1, TtNF-YA4B-1, TtNF-YA5 A-1, TtNF-YA5 A-2, TtNF-YA5 A, TtNF-YA5B-1, TtNF-YA5B-2, TtNF-YA6 A-1, TtNF-YA6B-1* in the *T. turgidum* genome based on their chromosomal location (Fig. 1). The SMART (http://smart.embl-heidelberg.de/) and InterProt (https://www.ebi.ac.uk/interpro/) databases revealed the existence of the PF02045 NF-YA domain. The identified *TtNF-YA* genes were distributed across eight chromosomes (2 A, 2B, 4 A, 4B, 5 A, 5B, 6 A and 6B). Their coding sequences varied from 726 to 1206 bp for *TtNF-YA5B-1* and *TtNF-YA4 A*, respectively. The distribution of the *TtNF-YA* genes on the eight chromosomes revealed that two genes are located on chromosome 4 A (*TtNF-YA4 A* and *TtNF-YA4 A-1*), three genes are located on chromosome 5 A (*TtNF-YA5 A-1, TtNF-YA5 A-2*, and* TtNF-YA5 A)*, two genes are located on chromosome 5B (*TtNF-YA5B-1* and* TtNF-YA5B-2*), and one gene is located on the rest of the chromosomes (Fig. 1). Detailed information about the *TtNF-YAs* genes is reported in Table 1.

**Table 1:** General information on *TtNF-YA* genes in durum wheat

Gene Name	Gene ID	Chr	Start Site	End Site	Length (bp)	CDS (bp)
*TtNF-YA2 A-1*	TRITD2 Av1G057050	2 A	128,562,166	128,564,575	2410	912
*TtNF-YA2B-1*	TRITD2Bv1G067630	2B	174,479,365	174,481,092	2375	903
*TtNF-YA4 A*	TRITD4 Av1G003820	4 A	7,312,479	7,335,864	23,386	1206
*TtNF-YA4 A-1*	TRITD4 Av1G195760	4 A	577,993,860	577,995,402	1543	810
*TtNF-YA4B-1*	TRITD4Bv1G010020	4B	24,461,319	24,463,156	1838	891
*TtNF-YA5 A-1*	TRITD5 Av1G012840	5 A	27,396,326	27,399,892	3567	1026
*TtNF-YA5 A-2*	TRITD5 Av1G018890	5 A	41,696,652	41,700,875	4625	942
*TtNF-YA5 A*	TRITD5 Av1G198400	5 A	533,343,733	533,346,437	2502	981
*TtNF-YA5B-1*	TRITD5Bv1G010590	5B	27,874,214	27,875,786	1573	726
*TtNF-YA5B-2*	TRITD5Bv1G019090	5B	53,124,037	53,128,122	4484	939
*TtNF-YA6 A-1*	TRITD6 Av1G200860	6 A	562,739,102	562,742,261	2502	798
*TtNF-YA6B-1*	TRITD6Bv1G196010	6B	615,290,239	615,293,350	3112	768

Chr: chromosome; CDS: coding sequence

### Characterization of TtNF-YA family members, subcellular localization, and three-dimensional structure prediction

**Fig. 2:**
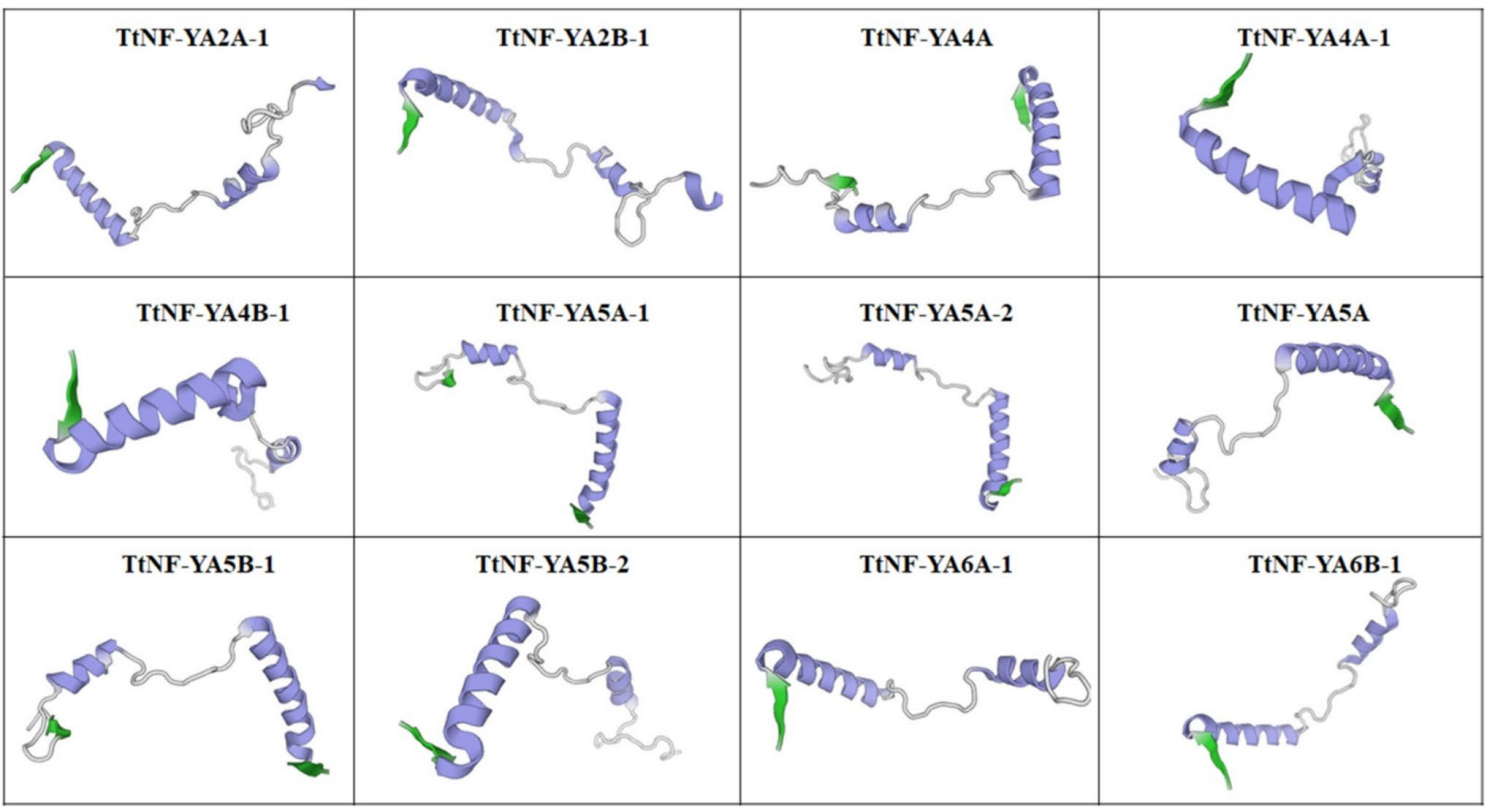
Putative 3D conformations of TtNF-YA proteins. The Swiss Model server was used to create these models. For the 3D structures of the TtNF-YA proteins, the secondary structure elements are represented as α-helices (purple), β-sheets (green), and coils (gray)

The proteins encoded by the *TtNF-YA* genes are variable in length, ranging from 241 (TtNF-YA5B-1) to 401 (TtNF-YA4 A) amino acids (Table 2). Their molecular weights ranged from 26.5 (TtNF-YA5 A-2) to 44.1 kDa (TtNF-YA4 A), and their calculated isoelectric points (IP) were between 7.13 (TtNF-YA4B-1) and 10.05 (TtNF-YA2B-1). Most of these proteins were found to be alkaline based on their predicted isoelectric points, with the only two neutral proteins being those encoded by TtNF-YA4B-1 and TtNF-YA5 A-2 (Table 2). The instability indices ranged from 49.32 (TtNF-YA5 A-1) to 60.51 (TtNF-YA4 A). Based on their predicted isoelectric points, most of TtNF-YA proteins were identified as alkaline, with the exception of TtNF-YA4B-1 and TtNF-YA5 A-2, which were found to be neutral (Table 2). The instability indices varied between 49.32 (TtNF-YA5 A-1) and 60.51 (TtNF-YA4 A). Additionally, all TtNF-YA proteins exhibited negative GRAVY values, suggesting their hydrophilic nature. Analysis using the BUSCA server revealed that all TtNF-YA proteins are localized in the nucleus. These findings, along with predictions of their three-dimensional structures, are summarized in Table 2 and illustrated in Fig. 2, respectively. The presence of α-helices (purple), β-leaflets (green), and coils (gray) was revealed in the TtNF-YA protein structures. Three of the twelve TtNF-YA proteins (TtNF-YA2 A-1, TtNF-YA2B-1 and TtNF-YA4B-1) had β-leaflets, and the remaining proteins, TtNF-YA4 A, TtNF-YA4 A-1, TtNF-YA5 A-1, TtNF-YA5 A-2, TtNF-YA5 A and TtNF-YA5B-1, had no β-sheets. The predicted 3D structures of the TtNF-YA proteins were classified as adaptable because they contained spirals.

**Table 2:** Physicochemical properties of *TtNF-YA* genes predicted in durum wheat

Gene Name	Protein (aa)	PI	MW (kDa)	II	GRAVY	Predicted localization
*TtNF-YA2 A-1*	303	9.62	33.118	53.71	−0.676	Nucleus
*TtNF-YA2B-1*	300	9.62	32.702	59.19	−0.728	Nucleus
*TtNF-YA4 A*	401	10.02	44.129	60.51	−0.748	Nucleus
*TtNF-YA4 A-1*	269	9.30	29.000	49.64	−0.857	Nucleus
*TtNF-YA4B-1*	296	7.13	32.227	55.87	−0.754	Nucleus
*TtNF-YA5 A-1*	341	8.65	36.938	49.32	−0.533	Nucleus
*TtNF-YA5 A-2*	313	7.23	34.224	55.12	−0.741	Nucleus
*TtNF-YA5 A*	326	9.20	35.077	52.25	−0.567	Nucleus
*TtNF-YA5B-1*	241	9.30	26.506	47.95	−0.685	Nucleus
*TtNF-YA5B-2*	312	8.85	34.491	59.14	−0.787	Nucleus
*TtNF-YA6 A-1*	265	9.78	29.044	52.81	−0.619	Nucleus
*TtNF-YA6B-1*	255	9.89	27.696	55.13	−0.698	Nucleus

*PI* isoelectric point, *MW* molecular weight (Da), *II* instability index, *GRAVY* Grand average of hydropathicity

### Phylogenetic analysis, motif conservation and protein–protein interactions of TtNF-YA family members

**Fig. 3:**
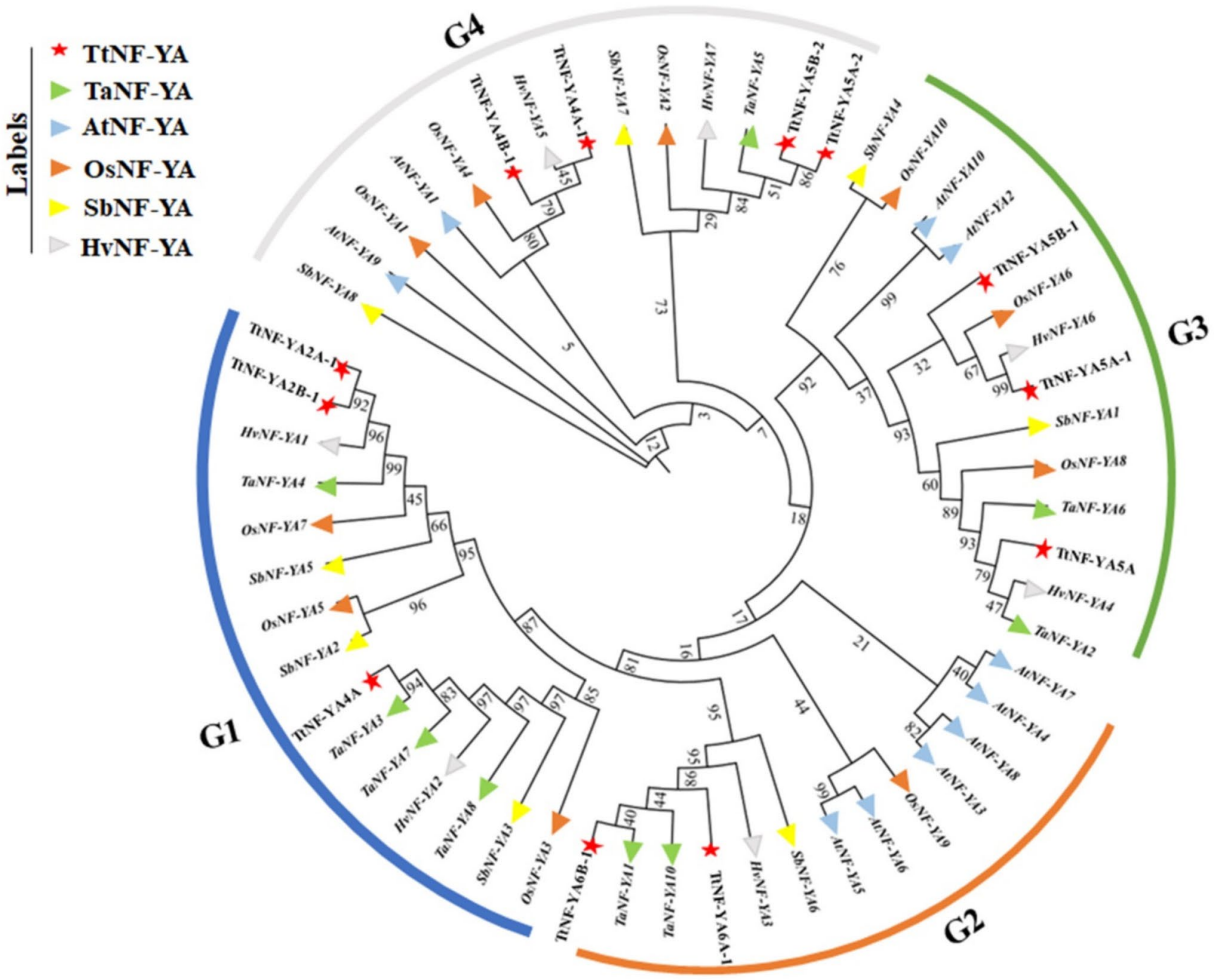
Evolutionary relationship of NF-YA proteins in *T. turgidum*, *T. aestivum*, *A. thaliana*, *O. sativa*, *S. bicolor*, and *H. vulgare* species. Using MEGA11 software with 1000 bootstrap repetitions. Proteins from *T. turgidum* are indicated by red stars, proteins from *T. aestivum* by green triangles, proteins from *A. thaliana* by blue triangles, and proteins from *O. sativa* by orange triangles. Proteins from *S. bicolor* are indicated by yellow triangles, and *H. vulgare* proteins are represented by gray triangles

**Fig. 4:**
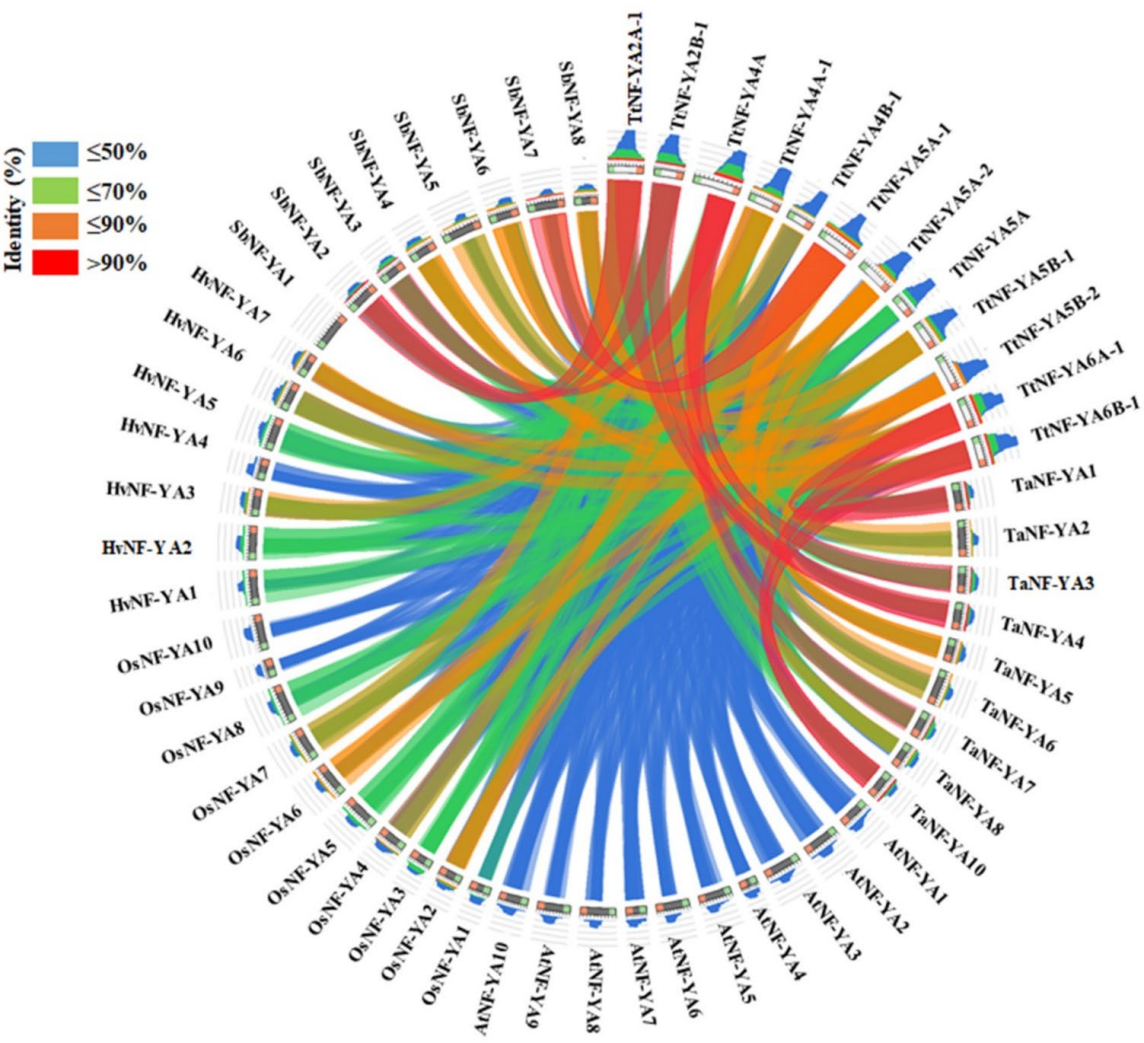
Synteny analysis of NF-YA members in the genomes of *T. turgidum*, *T. aestivum*, *A. thaliana*, *O. sativa*, *S. bicolor*, and *H. vulgare* using the Circoletto tool. The blue, green, orange and red colors represent ≤ 50%, ≤ 70%, ≤ 90% and > 90% identity, respectively

**Fig. 5:**
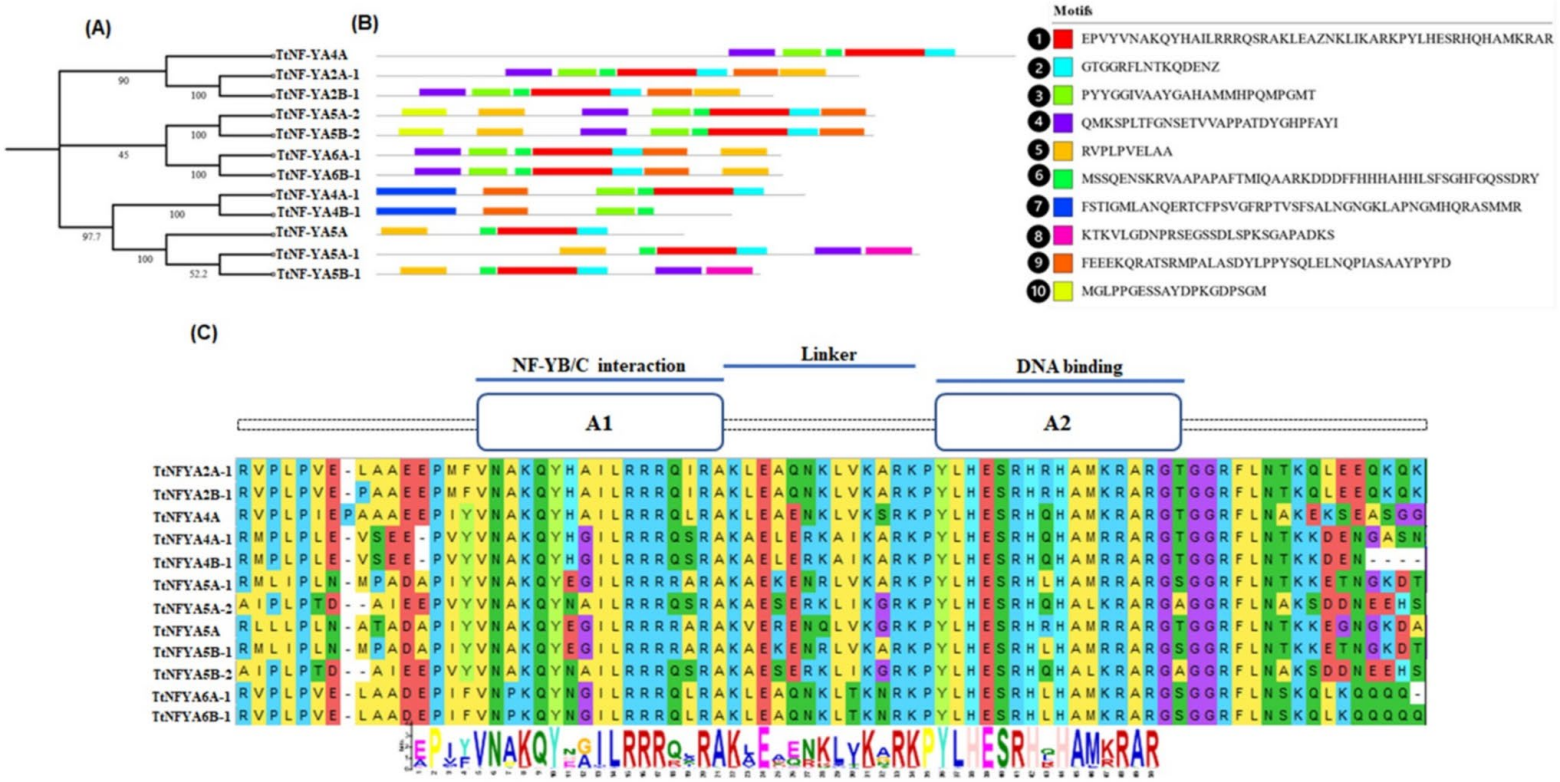
Structural analysis of *Triticum durum* TtNF-YA proteins. **A** Phylogenetic tree using TtNF-YA protein sequences. **B** Study of conserved motifs in TtNF-YA protein sequences. These motifs are shown in boxes of different colors. **C** Multiple alignments of conserved domains of the *TtNF-YA* gene family with DNA-binding and subunit interaction domains

**Fig. 6:**
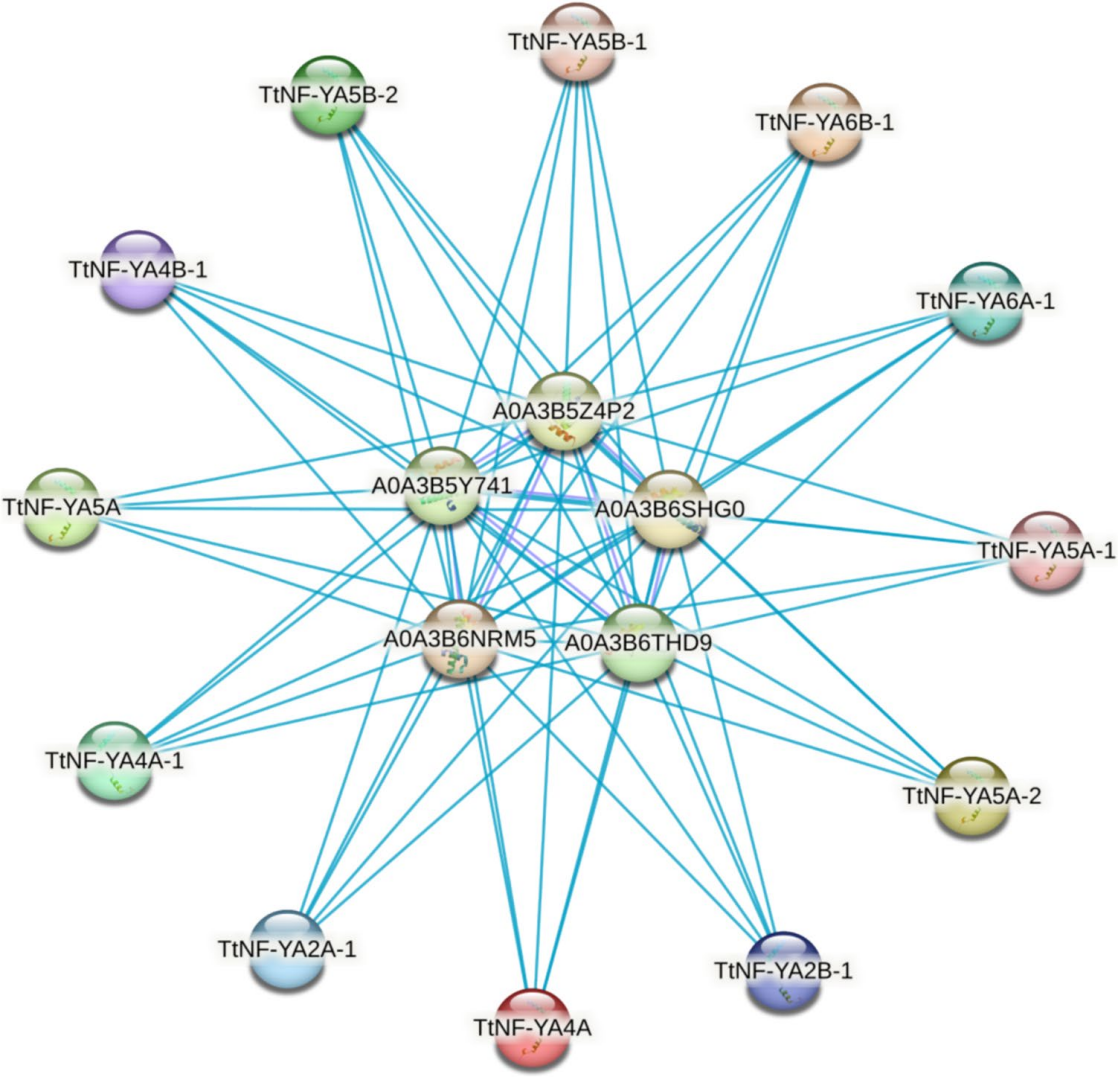
TtNF-YA proteins interaction network prediction using search tool for the retrieval of interacting genes/proteins (STRING)

Based on sequence similarities, four groups of NF-YA proteins were identified (Fig. 3). TtNF-YA4 A was orthologous to TaNF-YA3 and TtNF-YA6B-1 was orthologous to TaNF-YA1. The evolutionary relationship between TtNF-YA family genes in *T. turgidum* and those in other cereal species, including *T. aestivum*, *O. sativa*, *S. bicolor*, and *H. vulgare*, and the dicotyledonous model plant *A. thaliana* has been demonstrated through synteny analysis. The *TtNF-YA* genes of *T. turgidum* generally presented a syntenic network with the genes of these plant species (Fig. 4). Ten conserved motifs in TtNF-YA protein family members were found using the MEME program (Fig. 5A and B). As shown in Fig. 5B, the results demonstrated a high degree of similarity between protein sequences. All TtNF-YA proteins contained motif 1 and motif 6. Remarkably, motif 2 was present in most proteins except for TtNF-YA4B-1. On the other hand, motif 3 was found in most TtNF-YA members, with the exception of TtNF-YA5 A-1, TtNF-YA5 A and TtNF-YA5B-1, while motif 4 was present in TtNF-YA2 A-1, TtNF-YA2B-1, TtNF-YA4 A, TtNF-YA5 A-1, TtNF-YA5 A-2, TtNF-YA5B-1, TtNF-YA5B-2, TtNF-YA6 A-1 and TtNF-YA6B-1. In addition, motif 5 was present in the TtNFYA2B-1, TtNF-YA4 A, TtNF-YA5 A-1, TtNF-YA5 A-2, TtNF-YA5 A, TtNF-YA5B-1, TtNF-YA5B-2, TtNF-YA6 A-1, and TtNF-YA6B-1 proteins. Motif 7 was unique to TtNF-YA4 A-1 and TtNF-YA4B-1; in the same way, only TtNF-YA5 A-1 and TtNF-YA5B-1 possessed the motif 8. Except for TtNF-YA2 A-1, TtNF-YA5 A, TtNF-YA5 A-1, and TtNF-YA5B-1, most proteins had the motif 9, and TtNF-YA5 A-2 and TtNF-YA5B-2 shared the motif 10. The N- or C-terminal transcriptional regulatory domains of TtNF-YA proteins were found to be relatively variable, while some conserved regions were recognized based on multiple sequence alignments. The analysis revealed that the TtNF-YA conserved region has two domains, one for DNA binding (A2) and the other for the NF-YB/NF-YC interaction (A1) (Fig. 5C). The transcriptional heterotrimeric complex NF-Y is a composed of NF-YA, NF-YB, and NF-YC. The *in-silico* analysis of TtNF-YA protein–protein interaction network based on *T. aestivum* proteome experimental data revealed that all identified NF-YA in durum wheat may interact with the CBFD_NFYB_HMF domain containing proteins belonging to the TaNF-YB protein family. Thus, these findings suggest that the NF-YA proteins likely play a key role in the binding of the NF-YB subunit to the CCAAT box of eukaryotic promoters (Fig. 6).

### Gene organization and promoter region analyses

The architecture of exon-introns helps explain the evolutionary relationships between genes, as well as the evolutionary mechanisms that led to specific *TtNF-YA* gene structures in durum wheat. The *TtNF-YA* genes had three to six introns and four to seven exons, indicating that they may have the same RNA splicing pattern (Table 3 and Fig. S2). These results indicate that segmental duplication was responsible for the development of the *TtNF-YA4 A, TtNF-YA4 A-1, TtNF-YA5 A-1, TtNF-YA5 A-2, TtNF-YA5 A, TtNF-YA5B-1*, and *TtNF-YA5B-2* genes, while the other duplications are the result of whole genome duplication. All identified *TtNF-YA* genes exhibited high homology with their *T. aestivum* counterparts, including *TtNF-YA2B-1*‒*TtNF-YA5 A-2* and *TtNF-YA5B-2*. Further details are provided in Table 3.

**Table 3:** Study of duplication types of *TtNF-YA* genes and their orthologues in *T. aestivum*

Gene ID	Gene name	Exon number	Homologous	Paralogous	Duplication type	Orthologous (*T. aestivum*)
TRITD2 Av1G057050	*TtNF-YA2 A-1*	6	*TtNF-YA2B-1*	*TtNF-YA4 A*	WGD	*TaNF-YA4*
TRITD2Bv1G067630	*TtNF-YA2B-1*	6	*TtNF-YA2 A-1*	*TtNF-YA6B-1*	SD	*TaNF-YA4*
TRITD4 Av1G003820	*TtNF-YA4 A*	7	*Ø*	*TtNF-YA2 A-1*	SD	*TaNF-YA3*
TRITD4 Av1G195760	*TtNF-YA4 A-1*	6	*TtNF-YA4B-1*	*TtNF-YA2 A-1*	WGD SD	*TaNF-YA7*
TRITD4Bv1G010020	*TtNF-YA4B-1*	6	*TtNF-YA4 A-1*	*TtNF-YA2B-1*	SD	*TaNF-YA3*
TRITD5 Av1G012840	*TtNF-YA5 A-1*	5	*TtNF-YA5B-1*	*TtNF-YA4 A-1*	SD	*TaNF-YA6*
TRITD5 Av1G018890	*TtNF-YA5 A-2*	8	*TtNF-YA5B-2*	*TtNF-YA4 A-1*	WGD SD	*TaNF-YA5*
TRITD5 Av1G198400	*TtNF-YA5 A*	5	*Ø*	*TtNF-YA4 A-1*	SD	*TaNF-YA2*
TRITD5Bv1G010590	*TtNF-YA5B-1*	4	*TtNF-YA5 A-1*	*TtNF-YA2B-1*	WGD	*TaNF-YA6*
TRITD5Bv1G019090	*TtNF-YA5B-2*	8	*TtNF-YA5 A-2*	*TtNF-YA6B-1*	WGD SD	*TaNF-YA5*
TRITD6 Av1G200860	*TtNF-YA6 A-1*	5	*TtNF-YA6B-1*	*TtNF-YA4 A*	SD	*TaNF-YA10*
TRITD6Bv1G196010	*TtNF-YA6B-1*	5	*TtNF-YA6 A-1*	*TtNF-YA2B-1*	WGD	*TaNF-YA10*

Ø: Non-identified, WGD: whole genome duplication, SD: segmental duplication

**Fig. 7:**
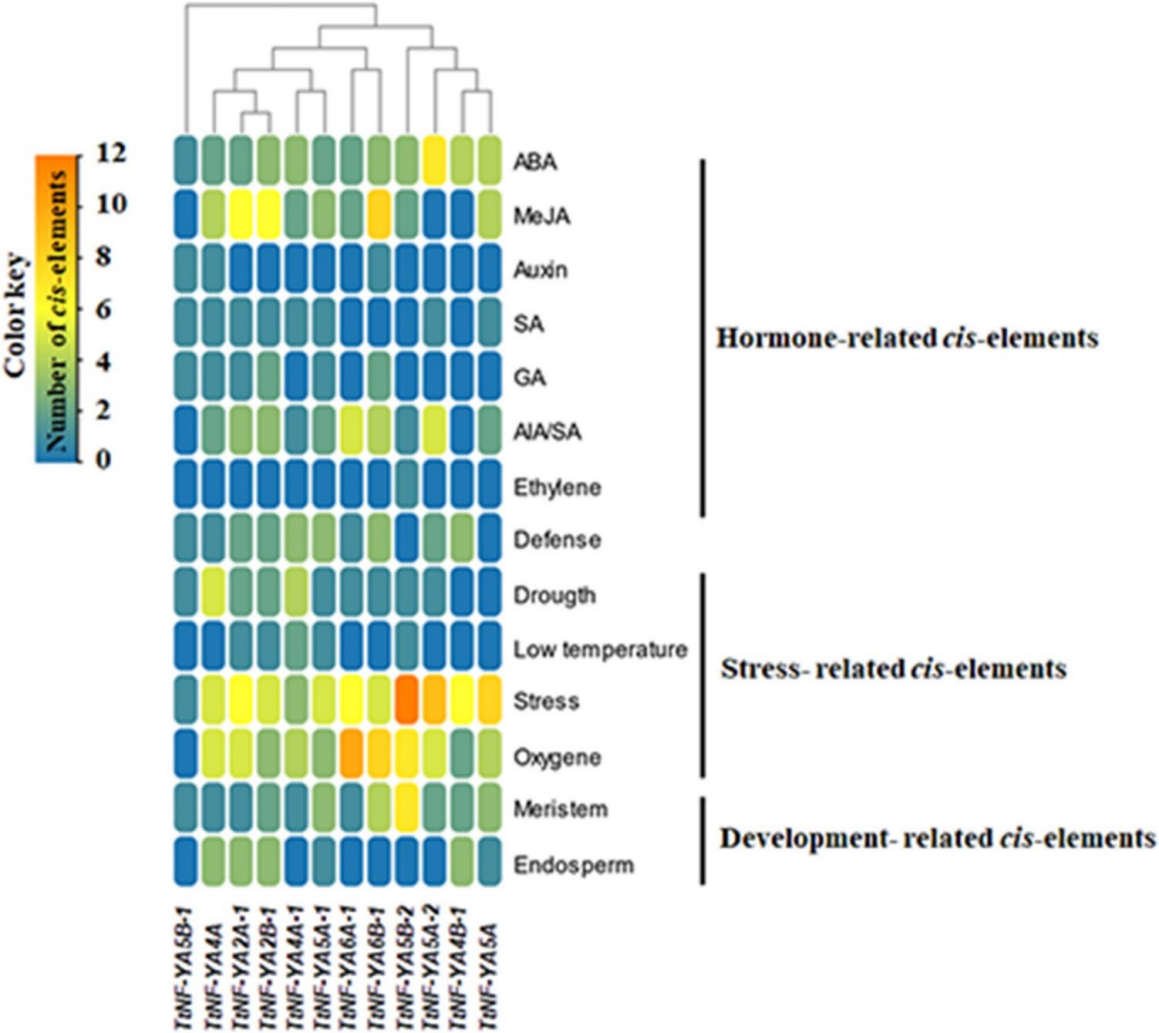
Heatmap showing prediction of cis-regulatory elements in the promoter regions of *TtNF-YA* genes

Information about gene function may be revealed by an analysis of gene promoter regions (Ben Saad et al. 2020; Huang et al. 2012). To understand the regulatory processes of *TtNF-YA* genes, an *in-silico* analysis of the 2 kb upstream sequence of those genes was conducted. Our primary objective was to identify the fundamental components, which were classified into three primary categories: hormone signaling, development, and stress responsiveness. Several cis-acting elements identified in these sequences were hormone binding sites for: ethylene (ERE), gibberellic acid (GA; GARE motifs and P box), salicylic acid (SA; TCA elements), auxin (IAA; AuxRR-core and TGA elements), methyl jasmonate (MeJA; CGTCA and TGACG motifs) and abscisic acid (ABA; ABRE elements) (Fig. 7). A wide range of stress-regulated *cis*-acting elements were also identified, including those related to low temperature (LTR), drought (DREs and MBS), plant defense (TC-rich motifs, W-box and WUN motif), hypoxia (O_2_-site) and other stresses, including heat shock, osmotic stress, and nutrient deprivation (STRE). In addition, some components were found to be associated with anaerobic respiration (ARE and GC motifs), meristem expression (CAT box and CCGTCC-box), endosperm gene expression (AAGAA-motif and GCN4-motif) and cell proliferation and differentiation (AP-1). Our analysis revealed that the *TtNF-YA* gene promoter regions contain multiple regulatory elements, suggesting that these genes play important roles in a variety of biological processes in durum wheat.

### TtNF-YA genes are highly expressed in developing seeds and induced by various stresses and phytohormones

**Fig. 8:**
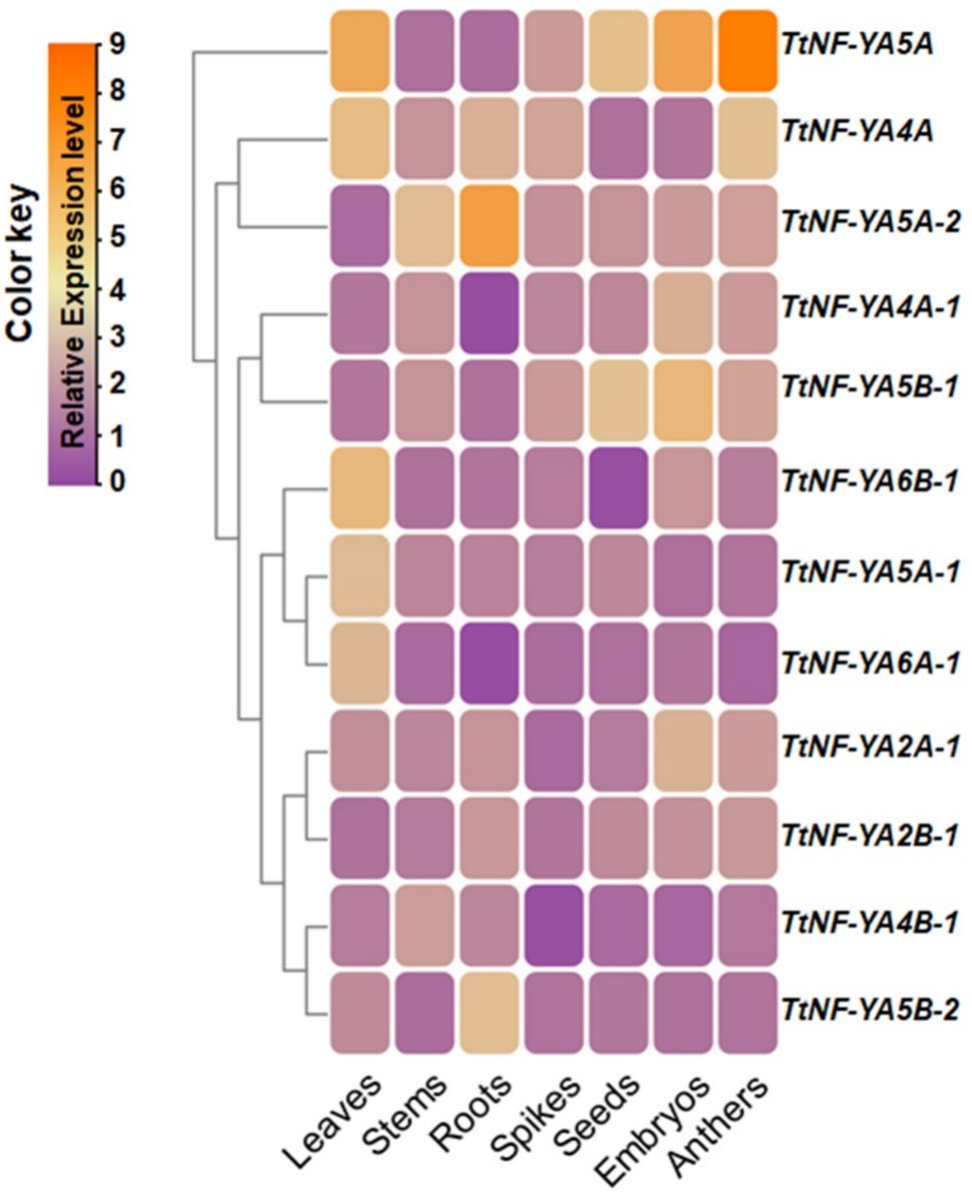
Heatmap of the expression pattern of *TtNF-YA* genes in seven durum wheat tissues. The *CDC* gene was used as an internal control. The data represent means of three independent experiments. Values in each cell are mean ± SEM (*n* = 3). Color code is presented on the left of the heatmap

To further investigate the roles and functions of twelve *TtNF-YA* genes in the durum wheat ‘Karim’, we sampled seven different tissues (roots, stems, leaves, spikes, anthers, seeds, and embryos) for the analysis of expression profile via RT-qPCR (Fig. 8). The data presented in Fig. 8 show the differential tissue-specific expression patterns of the TtNF-YAs genes. Among all the genes, TtNF-YA5 A-2 showed the highest expression in roots, while TtNF-YA5B-1 expression was markedly higher in embryos than in other tissues. Interestingly, TtNF-YA5 A expression significantly increased in leaves, seed embryos (21 days after anthesis), and anthers but not in roots or stems, indicating involvement of this gene in reproduction and seed development. These findings indicate that certain members of the *TtNF-YA* gene family may play a role in seed development in durum wheat, highlighting their potential significance in this important grain-yielding crop.

**Fig. 9:**
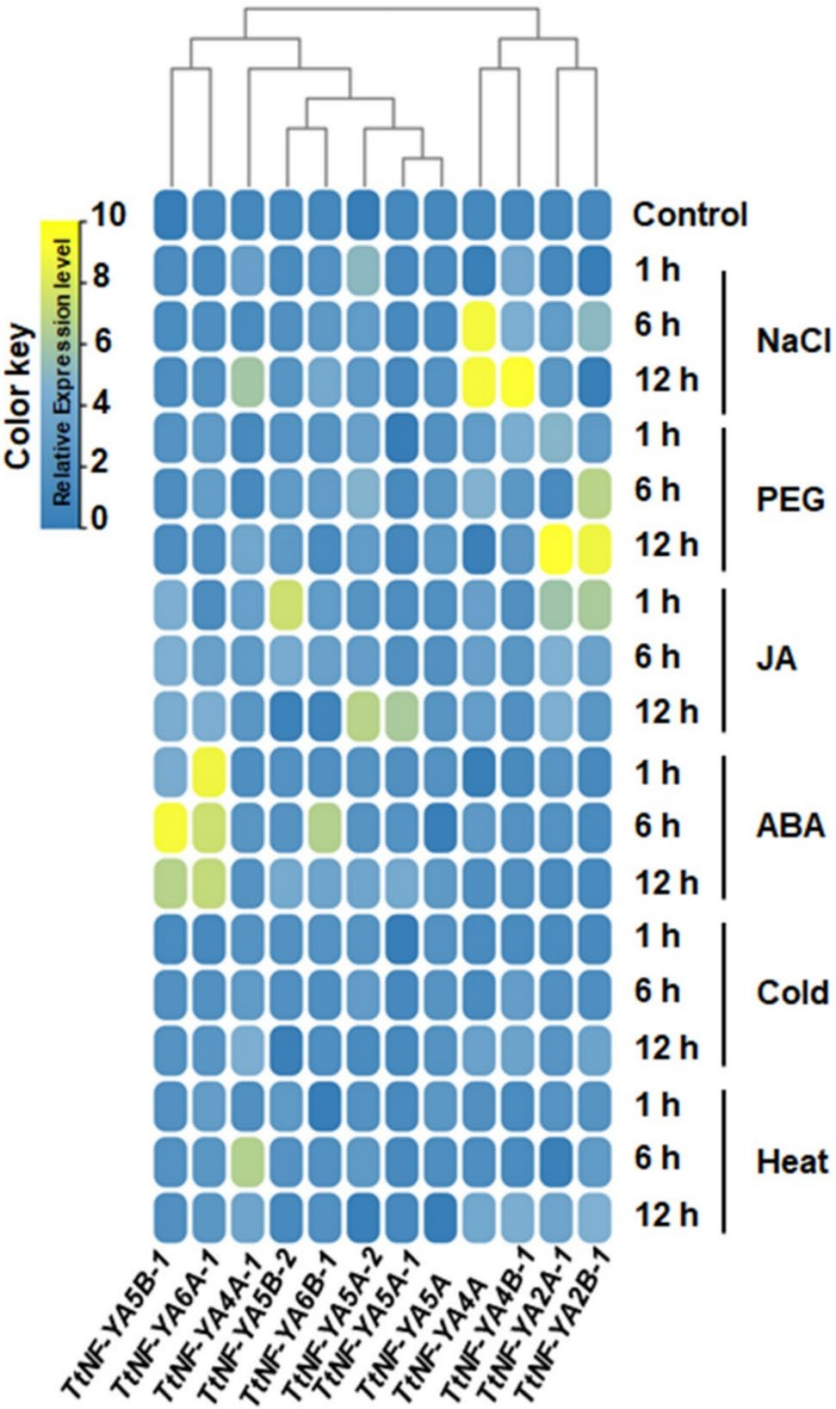
Analysis of the expression patterns of *TtNF-YA* genes in durum wheat plants exposed to 150 mM NaCl, 20% PEG-6000, 100 μM ABA, 100 µM MeJA, 4 °C cold, and 37 °C heat stress for 1, 6, and 12 h. The *CDC* gene was used as an internal control. Three plants were used per treatment per replicate

To better understand the impact of abiotic stresses and phytohormones on the expression of durum wheat ‘Karim’ *TtNF-YA* genes, we performed RT-qPCR analyses on seedlings exposed to these factors for 1, 6, and 12 h. As presented in Fig. 9, the expression of most *TtNF-YA* genes was induced by all treatments, except for the *TtNF-YA5B-1* and *TtNF-YA6 A-1* genes, which were up-regulated by the application of phytohormones. Additionally, the transcript of all *TtNF-YA* genes was induced early (1 h) after stress exposure (Fig. 9). The expression profiles after application of various stresses exhibited diverse patterns compared to those of the control. For instance, the greatest increase in salt- induced *TtNF-YA* expression was observed for the *TtNF-YA2 A-1, TtNF-YA2B-1, TtNF-YA4 A, TtNF-YA4 A-1*, *TtNF-YA4B-1,* and *TtNF-YA5 A-2* genes 1 h after treatment, except for *TtNF-YA*6B-1, whose expression was induced after 12 h suggesting that the aforementioned genes play a role in salt tolerance mechanisms. The *TtNF-YA4 A-1* gene exhibited the highest expression under osmotic, cold and heat stress after 1 h of treatment compared to the other *TtNF-YA* genes. Treatment with ABA and MeJA for 12 h caused clear upregulation of *TtNF-YA5 A-1*, *TtNF-YA5 A-2*, *TtNF-YA5 A*, *TtNF-YA5B-1*, *TtNF-YA5B-2*, *TtNF-YA6 A-1*, and *TtNF-YA6B-1* genes. In contrast, these genes exhibited only minimal induction following exposure to other stress conditions (Fig. 9). This suggests that they may not necessarily be major contributors to the signaling pathways affected by the tested stressors. In addition, we demonstrated that most of *TtNF-YA* genes exhibited high induction levels under cold stress treatment for 6 h, except for the *TtNF-YA5 A-1* gene, which displayed a similar response to the control conditions. Similarly, under heat stress, the expression levels of six *TtNF-YA* genes (*TtNF-YA2 A-1, TtNF-YA2B-1, TtNF-YA4 A, TtNF-YA4 A-1*, *TtNF-YA4B-1, TtNF-YA5 A-2*, *TtNF-YA5 A*,* TtNF-YA6 A-1* and* TtNF-YA5B-2*) were significantly upregulated, except for *TtNF-YA5 A-1, TtNF-YA5B-1*, and *TtNF-YA6B-1* genes, which displayed similar responses or slight induction in comparison with the control (Fig. 9). Notably, the transcript levels of three genes (*TtNF-YA2 A-1, TtNF-YA2B-1,* and *TtNF-YA4 A-1*) were greater than those of the other genes under osmotic stress, while the *TtNF-YA5 A-2* and *TtNF-YA5B-1* genes did not respond to this stress at all (Fig. 9). Our findings revealed that the expression of the six *TtNF-YA* genes (*TtNF-YA2 A-1, TtNF-YA2B-1, TtNF-YA4 A, TtNF-YA4 A-1*, *TtNF-YA4B-1, TtNF-YA5 A-2*, and *TtNF-YA5B-2*) increased consistently following salt, osmotic, cold and heat stress treatments.

### Overexpression of TtNF-YA genes promotes stress tolerance in yeast cells

**Fig. 10:**
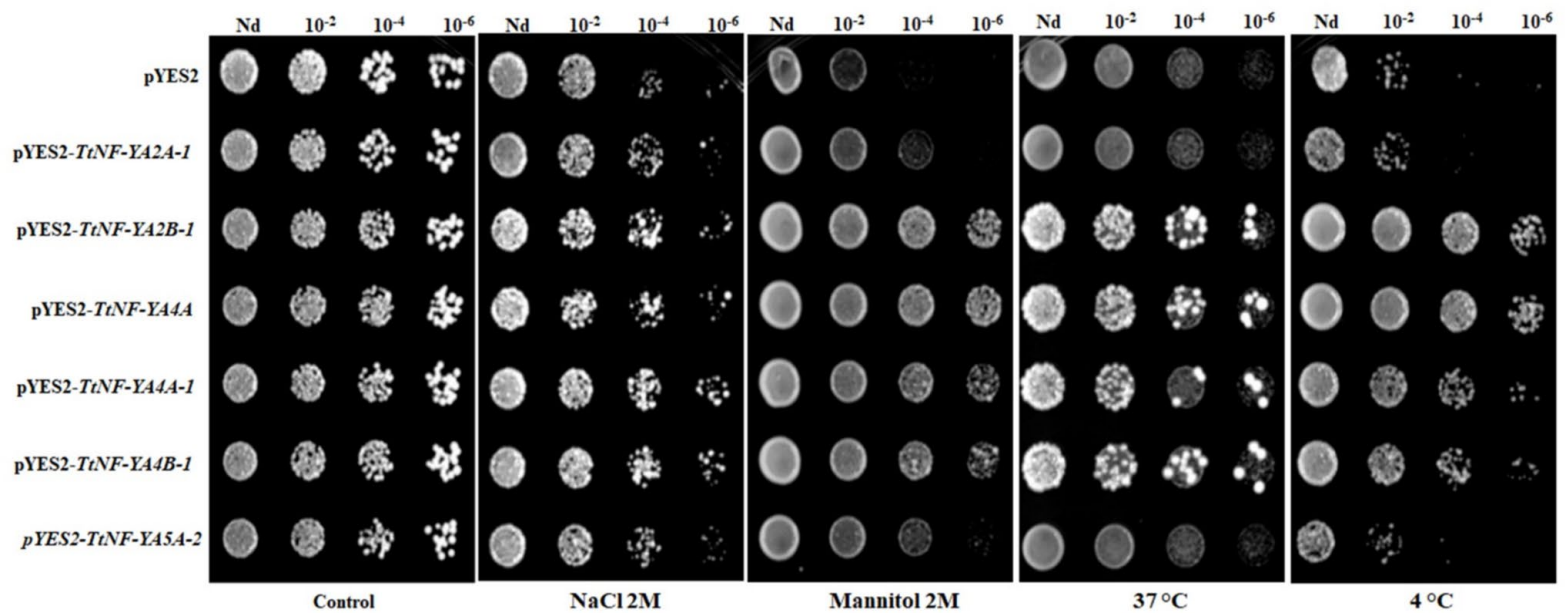
The heterologous expression of six *TtNF-YA* genes (*TtNF-YA2 A-1*, *TtNF-YA2B-1*, *TtNF-YA4 A*, *TtNF-YA4 A-1*, *TtNF-YA4B-1*, and *TtNF-YA5 A-2*) genes confers stress tolerance to yeast cells

Considering the fact that the twelve *TtNF-YA2* genes were upregulated under different abiotic stresses, we sought to determine the potential impact of TtNF-YA proteins in protecting yeast cells from abiotic stress using this heterologous model system. Based on the aforementioned RT-qPCR analysis, six *TtNF-YA* (*TtNF-YA2 A-1, TtNF-YA2B-1, TtNF-YA4 A, TtNF-YA4 A-1*, *TtNF-YA4B-1,* and *TtNF-YA5 A-2*), were chosen to investigate how their overexpression influences *S. cerevisiae* growth under stress conditions*.* For this purpose, we used transgenic yeast harboring pYES2-*TtNF-YA* vector, in which six genes (*TtNF-YA2 A-1*, *TtNF-YA2B-1*, *TtNF-YA4 A*, *TtNF-YA4 A-1*, *TtNF-YA4B-1*, and *TtNF-YA5 A-2*) were placed under the control of the galactose-inducible promoter. The tolerance of yeast cells to different abiotic stresses (salt, osmotic stress, cold, and heat) in solid media was evaluated after 3 days of exposure. As shown in Fig. 10, comparable growth patterns were observed between the yeast cells harboring *TtNF-YA* genes and those harboring the empty vector (EV), under control conditions. However, when yeast cells were exposed to stress factors, the growth of pYES2-*TtNF-YA* yeast cells was significantly greater than that of EV yeast cells (Fig. 10). In fact, under osmotic, heat and cold stress, yeast cells overexpressing *TtNF-YA2B-1*,* TtNF-YA4 A*,* TtNF-YA4 A-1* or *TtNF-YA4B-1* exhibited greater growth compared to cells overexpressing *TtNF-YA2 A-1* and *TtNF-YA5 A-2* construct or EV. Under salt condition, however, yeast cells transformed with the six *TtNF-YA* genes were capable of growing more effectively than the control strain (EV).

## Discussion

The NF-Ys are important transcription factors involved in plant growth, development, and environmental responses (Yan et al. 2013; Liu et al. 2016; Xu et al. 2016; Yu et al. 2020). Although the number of these genes and their encoded proteins have been characterized in various plant species, to date, durum wheat has yet to undergo studies on this aspect. Due to the large variability and number of NF-Ys in plants, before further study of the biological function of the TtNF-Y proteins in durum wheat, it is crucial to provide comprehensive overview of the *TtNF-Y* gene family, focusing on the gene structures, their homology, and tissue- and stress-specific expression profiles. Our study covered selected data on cis*-*elements and expression studies on this class of TFs as well as open-source data mining for expression analyses to provide insights into the role of the TtNF-YA subunit in various metabolic pathways in the durum wheat ‘Karim’. We characterized a total of twelve *TtNF-YAs* members in the durum wheat genome compared to other cereal crops: ten *NF-YA* in *T. aestivum* (Stephenson et al. 2007), fourteen *ZmNF-YA* in maize (Zhang et al. 2016), eleven *OsNF-YA* in rice (Yang et al. 2017c), and eight *SbNF-YAs* in sorghum (Malviya et al. 2016). In the model species *A. thaliana*, ten *AtNF-YA* were identified (Siefers et al. 2009), followed by extensive analyses in other crop plants, including legumes (Quach et al. 2015; An et al. 2022), vegetables (Li et al. 2016; Yan et al. 2021; Feng et al. 2023), fruit crops (Ren et al. 2016; Li et al. 2019; Qu et al. 2020), and ornamental and medicinal species (Wei et al. 2020; Wang et al. 2019), as well as in woody plants (Guo et al. 2021; Liu et al. 2021a). The number of *NF-YA* members identified to date in these species’ ranges from six *PpNF-YA*s in peach (Li et al. 2019) to twenty-one *GmNF-YA* in soybean (Quach et al. 2015).

The conserved regions involved in subunit interaction and DNA binding were found in TtNF-YA proteins, as demonstrated by multiple alignments, and these regions were also present in other plants (Wei et al. 2020; Panahi et al. 2019; Liu et al. 2021b). Despite this structural similarity, TtNF-YA proteins were characterized by different isoelectric points, molecular weights, instability index, and GRAVY values, which were observed in several previous studies on maize (Lv et al. 2022), and sorghum. In silico analysis of TtNF-YA proteins interaction networks based on *T. aestivum* proteome revealed that all TtNF-YA proteins interactors harbored the CBFD_NFYB_HMF domain and belonged to the NF-YB protein family. These findings suggest that NF-YA proteins likely play a key role in the binding of the NF-YB subunit to the CCAAT box present in eukaryotic promoters. However, further analysis using yeast two‐hybrid and bimolecular fluorescence complementation could help to discover the interactors and decipher the action mode of NF-YA proteins. Furthermore, the subcellular localization of the TtNF-YA proteins was predicted to be in the nucleus by in silico analysis, which was consistent with the studies on maize and *Saccharum* spp. (Lv et al. 2022; Swathik Clarancia et al. 2023). The intersections of NF-YA syntenic members between *T. turgidum*, *T. aestivum*, *O. sativa*, *S. bicolor*, *H. vulgare,* and model species *A. thaliana* may be useful for evolutionary research, as they may share expression patterns and important functional properties. Our results agree with those of previous research on the evolutionary relationship of *ShNF-Y* gene family with their homologs in sugarcane, *Arabidopsis*, and sorghum (Song et al. 2022).

Given the well-established link between cis-acting elements and gene regulation (Feng et al. 2023; Song et al. 2022), we investigated the presence of putative regulatory elements within the predicted promoter regions of each *TtNF-YA* gene. The analysis revealed that most *TtNF-YA* promoters harbored diverse cis-elements associated with various biological processes in plants. These included elements linked to growth and development, hormonal signaling, and stress responses such as drought tolerance, defense mechanisms, and wound healing. Most of identified *TtNF-YA* genes had a high number of ABA-responsive cis-elements, such as ABRE (*cis*-acting element involved in the abscisic acid responsiveness), which is essential for both abiotic stress tolerance and ABA signaling (Bailly et al. 2008). Similarly, the presence of drought-related cis-elements (MBS and DREs) in TtNF-YAs implies that they could play a role in drought resistance. Abscisic acid (ABA), ethylene (ET), and salicylic acid (SA) responsive elements were also discovered in the promoter regions of the *TtNF-YA* genes. These hormones are crucial elements of the stress responses in plants and interact in a unique way with the JA pathway to regulate the activation of a variety of effective defenses (Pauwels et al. 2009; Pieterse et al. 2009). Other *cis*-acting elements were also found, including motifs connected to the meristem and endosperm, which suggests that these genes might be involved in the development of various plant organs and that their expression may occur in specific tissues. There is evidence on *AtNF-Y* expression in the shoot apical meristem and its role in leaf formation (Zhang et al. 2017), as well as on the controlling role of *OsNF-Ys* in starch synthesis during endosperm formation in rice (Feng et al. 2022). We showed that *TtNF-YA* genes are involved in both vegetative and reproductive growth of shoots, and in seed development. Our findings were consistent with those reported in previous studies, for instance, on the peanut *AhNF-Y* genes, most of which were expressed in tissue-specific manner (Wan et al. 2021). Recently, Li et al. (2021) analyzed the expression patterns of all *StNF-Y* members in a series of potato organs and tissues and proved that the genes that are evolutionally related exhibit similar expression profiles, suggesting their involvement in corresponding developmental phenomena. Many studies have provided mechanistic evidence that *NF-Y* TFs play crucial functions in the regulation of flowering time and seed development in plants. Zhao et al. (2017) demonstrated that regulation of seed development by *AtNF-YB9* involved the integration of both light and hormonal signals, while the homologous gene *AtNF-YB6* modulated ABA signaling pathway, affecting embryo morphogenesis. Also, An et al. (2022) suggested the importance of four alfalfa *MsNF-YB* genes in seed development. In our study, we identified two genes associated with reproduction and seed development in durum wheat, *TtNF-YA6 A-1* and *TtNF-YA6B-1*, which could be of great importance in future breeding programs of this crop.

Considering crucial role of *NF-Y* TFs family in stress responses in plants (Myers and Holt 2018), we highlighted that the expression of most of the identified *TtNF-YAs* genes was influenced by salinity, osmotic stress, temperature stress (both cold and heat), and the application of stress-related phytohormones (ABA and MejA). In particular, the intricate expression patterns of *TtNF-YAs* genes observed after phytohormone treatments suggest their potentially integrative role during plant growth and development. *NF-YA1* and *NF-YA9* genes from *Saccharum hybrid* and *Erianthus arundinaceus*, respectively, were down-regulated in the leaf and root tissues under drought conditions. Studies have shown tissue-specific expression patterns for *NF-YA* genes. For example, *NF-YA3* exhibited higher expression in *E. arundinaceus* roots, while *NF-YA5* expression was elevated in both leaf and root tissues of *E. arundinaceus* and a *Saccharum hybrid* (Swathik Clarancia et al. 2023). Several studies have also linked *NF-YA* genes to stress tolerance. In *Arabidopsis*, *AtNF-YA2, AtNF-YA3*, and *AtNF-YA5* are implicated in drought tolerance (Laloum et al. 2013). Similarly, homologs in other plant species demonstrate stress-protective roles. For instance, *StNF-YA9* (paralog of *AtNFY-A1*) is upregulated by various stresses and regulates post-germination growth arrest under salt stress conditions (Li et al. 2013). Transgenic rice expressing *OsNF-YA7* exhibits improved drought tolerance (Yang et al. 2017c), and *PtNF-YA9* overexpression in *Arabidopsis* enhances drought resistance by preventing excessive growth after germination, thus protecting *Populus trichocarpa* seeds from water scarcity (Lian et al. 2018). Additionally, *NF-YAs* contribute to drought resistance in soybean (Yu et al. 2020). Wang et al. (2023) in their previous work identified significant expression changes in *GbNF-YA* genes from *Ginkgo biloba* under heat, drought, and salt stress with *GbNF-YA6* specifically upregulated by both heat and drought (Wu et al. 2018; Li et al. 2013; Ma et al. 2015b).

To gain deeper insight into the role of *TtNF-YA* genes in stress tolerance and test their ability to induce such tolerance, *TtNF-YA2 A-1/2B-1/4 A/4 A-1/4B-1* and *5 A-2* were overexpressed in yeast cells, as *S. cerevisiae* grows rapidly and can be cultured inexpensively on simple media (Tullio 2022) and is genetically easy to manipulate (Stepchenkova et al. 2023). Yeast provides a straightforward eukaryotic system for analyzing gene functions related to protein interactions and stress responses (Tullio 2022). Although it lacks NF-YA genes, its Hap complex serves a similar role to the NF-Y complex in higher eukaryotes, making it suitable for preliminary studies. The insights gained from such experiments can guide further research in model plants like *Arabidopsis thaliana* and prioritize targets for transgenic studies in durum wheat, ultimately bridging findings to agricultural applications. The growth of *TtNF-YA*-transformed yeast cells was enhanced under the tested abiotic stress conditions, suggesting that *TtNF-YAs* are functional in yeast and actively ameliorate the effects of stressful environmental conditions. Furthermore, our results indicate that in the yeast proteome, protein partners to *TtNF-YA* exist, allowing for the preliminary assumption that some stress response pathways may be shared between plants and yeasts. Although exploitation of yeast as a model system has limitations and may not fully represent durum wheat responses, understanding the mechanisms in which *TtNF-YAs* genes confer stress tolerance in this simplified model of eukaryotic cells may facilitate deciphering of the functional roles of *TtNF-YAs* in stressed durum wheat and understanding the universal molecular links that contribute to stress tolerance in plants and fungi.

## Conclusions

**Fig. 11:**
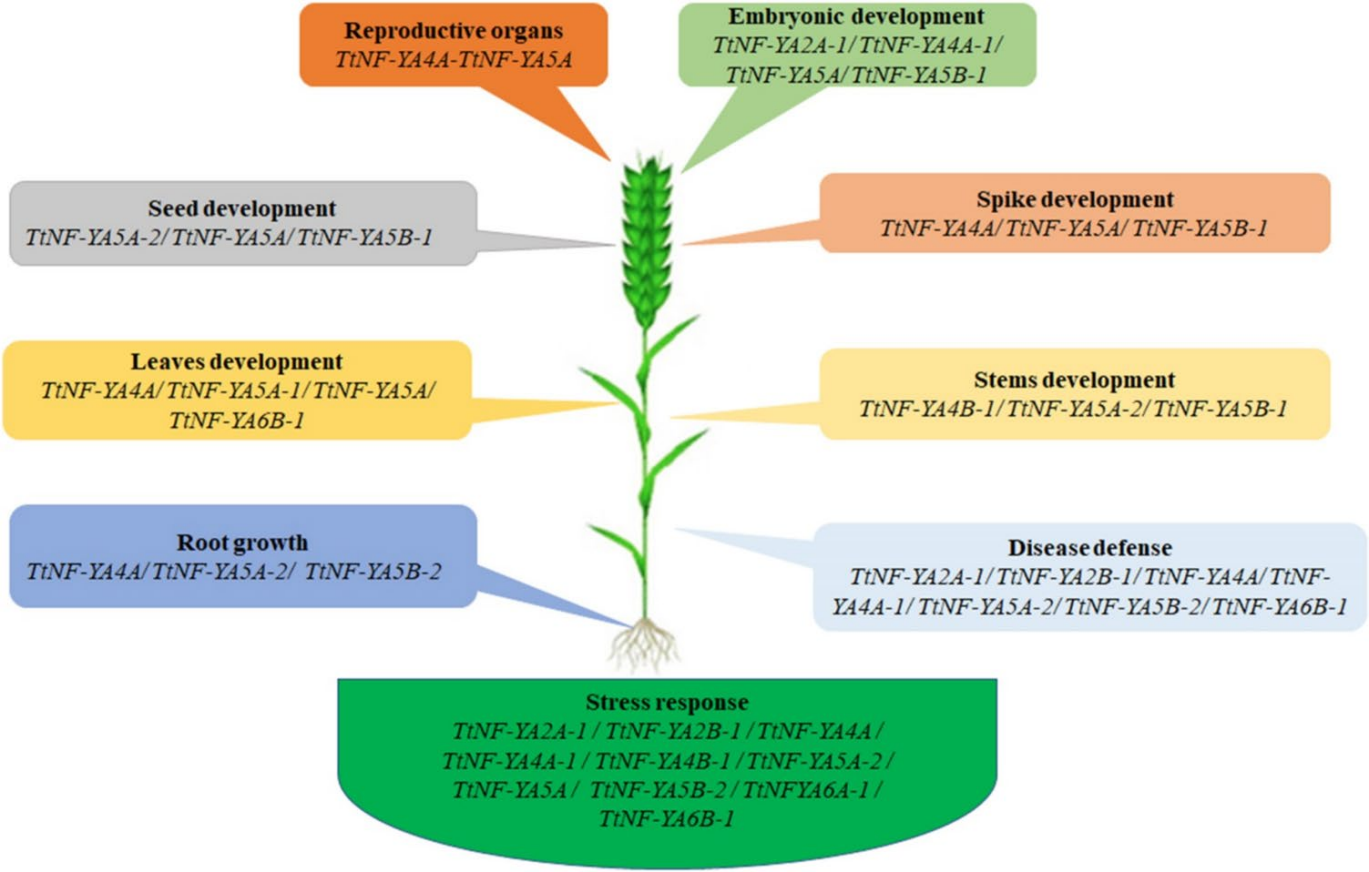
Schematic presentation of the probable functions of durum wheat *TtNF-YA* genes. Based on the tissue/organ expression patterns and qRT-PCR data under stress, the probable biological processes involving TtNF-YAs were summarized

In this study we provide the first comprehensive overview of *TtNF-YAs* genes in durum wheat according to the tissue/organ expression patterns and the RT-qPCR analysis under versatile stress conditions (Fig. 11). The likely biological processes in which *TtNF-YAs* are involved have been summarized based on tissue/organ expression profiles and stress qRT-PCR data. *TtNF-YAs* are involved in the development of vegetative and reproductive systems, including the growth of spikes, roots, seeds, leaves, stems, and embryonic development. Moreover, *TtNF-YAs* contribute to the development of reproductive organs, disease defense, and reaction to stress. We identified genes involved in both vegetative and reproductive growth that play a role in the abiotic stress response and disease resistance. Twelve *TtNF-YA* genes were found in total, and information about their structure, localization, phylogenetic characterization, tissue-specific expression patterns, and expression profiling under abiotic stress conditions was gathered. The significant role of six *TtNF-YA* genes in abiotic stress tolerance was also confirmed by the study of their overexpression in yeast cells. Overall, our results provide new insights into the roles of *TtNF-YA* genes and proteins, which could facilitate breeding towards more stress-resilient crops.

## Supplementary Information

Below is the link to the electronic supplementary material.

Supplementary file1 (DOCX 612 KB)

Supplementary file2 (DOCX 22 KB)

## Data Availability

No datasets were generated or analysed during the current study.
