# Supplementary material for: Durum wheat nuclear factor Y (NF-Y) a subfamily: structure, phylogeny, and expression analysis in response to hormones and abiotic stresses
Source: Funct Integr Genomics. 2025 May 14;25(1):102. doi: 10.1007/s10142-025-01607-z (PMC12075364; doi:10.1007/s10142-025-01607-z)
Supplement: Supplementary file 1 — Supplementary file1 (DOCX 612 KB) [file 10142_2025_1607_MOESM1_ESM.docx]

**SUPPLEMENTARY MATERIALS**


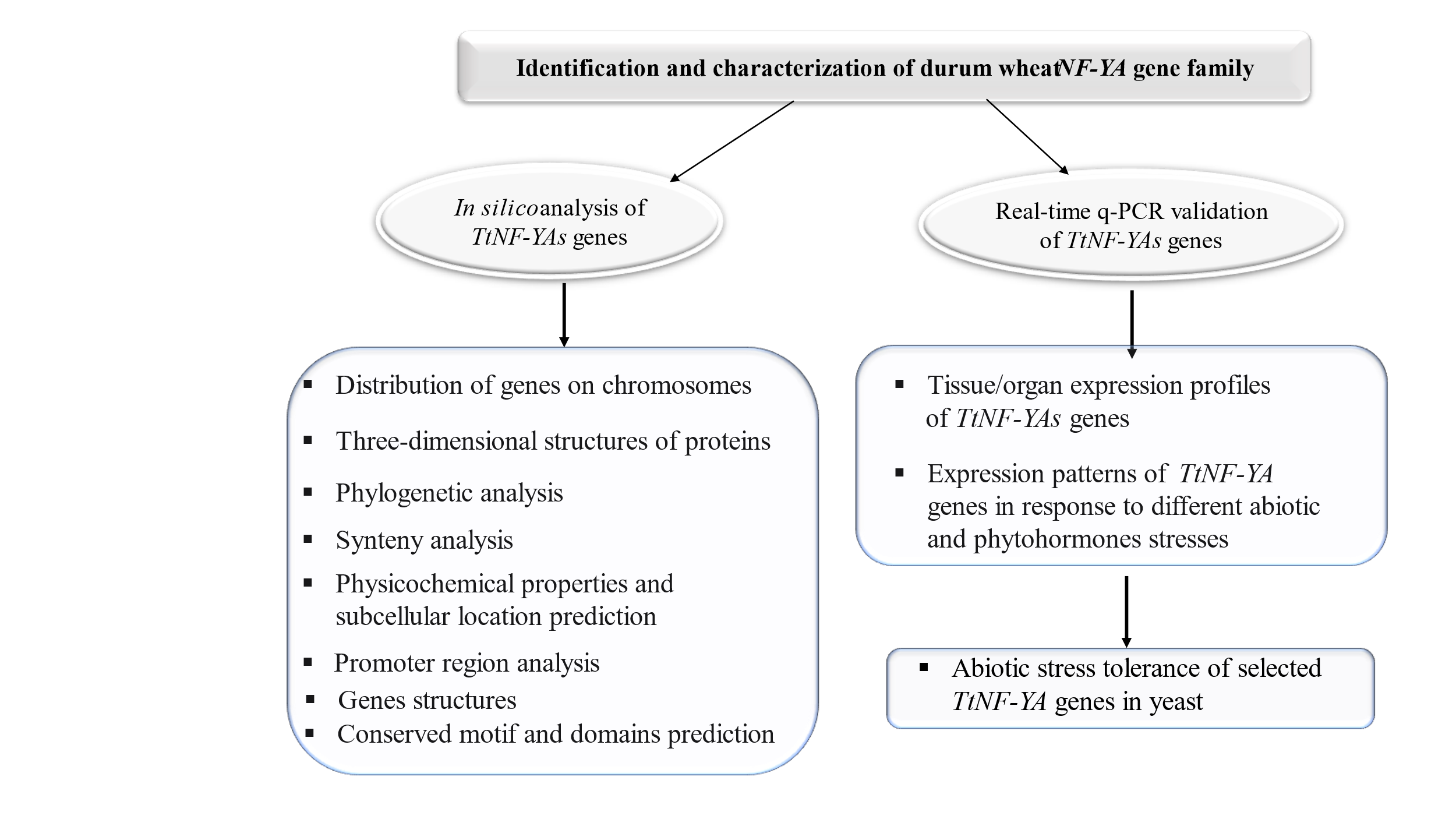


**Fig. S1** Flow chart of the work


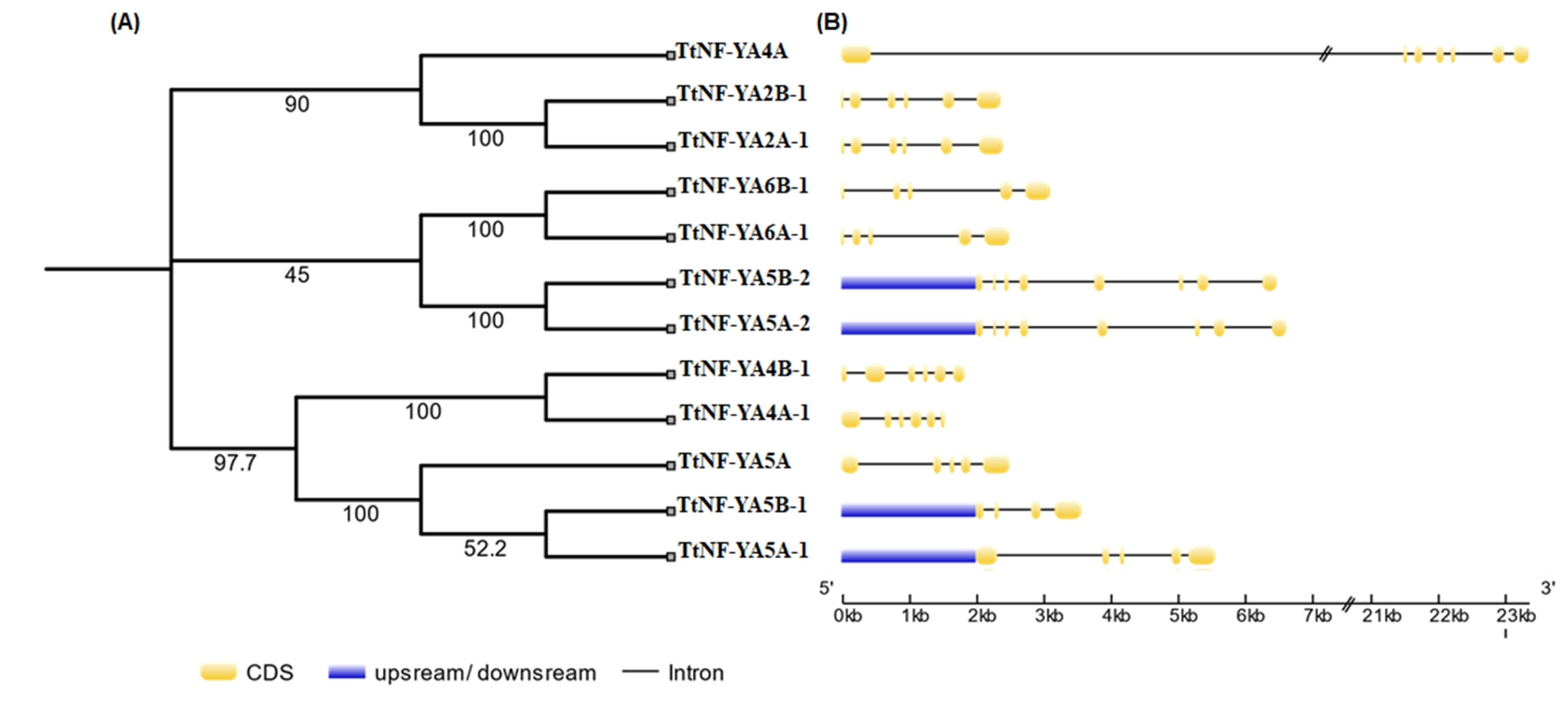


**Fig. S2.** Analysis of twelve *TtNF-YA* gene structures. (A) Phylogenetic tree using *TtNF-YA* gene sequences. (B) Observation of the intron/exon structures of the TtNF-YA family.
