# Supplementary material for: Durum wheat nuclear factor Y (NF-Y) a subfamily: structure, phylogeny, and expression analysis in response to hormones and abiotic stresses
Source: Funct Integr Genomics. 2025 May 14;25(1):102. doi: 10.1007/s10142-025-01607-z (PMC12075364; doi:10.1007/s10142-025-01607-z)
Supplement: Supplementary file 2 — Supplementary file2 (DOCX 22 KB) [file 10142_2025_1607_MOESM2_ESM.docx]

**Table S1** Sequences of primers used in PCR and RT-qPCR analysis.

| Primers | Sequences |
| --- | --- |
| *qTtNF-YA2A-1-F* | 5’-TTGAGCTTGCAGCAGAAGAG-3’ |
| *qTtNF-YA2A-1-R* | 5’- AGCTTATTTTGGGCCTCCAG-3’ |
| *qTtNF-YA2B-1-F* | 5’-CCGATCAGCACATGGTAAAG-3’ |
| *qTtNF-YA2B-1-R* | 5’-ATCCCAGCATAAGCATCAGC-3’ |
| *qTtNF-YA4A-F* | 5’-ATCGCGATGGCTATACGAAG-3’ |
| *qTtNF-YA4A-R* | 5’-CGGTGTGTATGCCATTTCTG-3’ |
| *qTtNF-YA4A-1-F* | 5’-GAGATCAGGGGAACCAAAAG-3’ |
| *qTtNF-YA4A-1-R* | 5’-GAAGCCATCGGTTGGTTTAG-3’ |
| *qTtNF-YA4B-1-F* | 5’-GCCTGAGATTTGCTTCCTTG-3’ |
| *qTtNF-YA4B-1-R* | 5’-CGATCGGTTGGTTTAGTTCC-3’ |
| *qTtNF-YA5A-1-F* | 5’-CTGAATATGCCTGCTGATGC-3’ |
| *qTtNF-YA5A-1-F* | 5’-TGGCCTTGACCAACCTATTC-3’ |
| *qTtNF-YA5A-2-F* | 5’-ACCCAACTGTCGATCCATTC-3’ |
| *qTtNF-YA5A-2-R* | 5’- CATTGGTGGGTGCATCATAG-3’ |
| *qTtNF-YA5A-F* | 5’-TGCTTACCACTTACGCGATG-3’ |
| *qTtNF-YA5A-R* | 3’-CATACTGCTTCGCATTCACG-3’ |
| *qTtNF-YA5B-1-F* | 5’-ACTGAACATGCCTGCTGATG-3’ |
| *qTtNF-YA5B-1-R* | 5’-TATGGCTTTCTGGCCTTGAC-3’ |
| *qTtNF-YA5B-2-F* | 5’-TTCCAGCTCCAAAGACAAGC-3’ |
| *qTtNF-YA5B-2-R* | 3’-TCATGGTTCCCCGACTTATC-3’ |
| *qTtNF-YA6A-1-F* | 5’-CCGTTCAACGATTCTGGTTC-3’ |
| *qTtNF-YA6A-1-R* | 5’-AACTCTTGCCGTTGCAGATG-3’ |
| *qTtNF-YA6B-1-F* | 5’-CCGTTCAACGATTCTGGTTC-3’ |
| *qTtNF-YA6B-1-R* | 5’-TCATCCGCTGCTAATTCGAC-3’ |
| *qCDC-F* | 5’-GCCTGGTAGTCGCAGGAGGAT-3’ |
| *qCDC-R* | 5’-ATGTCTGGCCTGTTGGTAGC-3’ |
| *TtNF-YA2A-1-EcoR*I | 5’-TTAGAATTCATGTGTAAAATGGAGGATCA-3’ |
| *TtNF-YA2A-1-Xba*I | 5’-TTATCTAGATTACCTCATCATGGAAGCCC-3’ |
| *TtNF-YA2B-1-EcoR*I | 5’- TTAGAATTCATGCGATTATATCTTCATGC-3’ |
| *TtNF-YA2B-1-Xba*I | 5’- TTATCTAGATTACCTCATCATGGAAGCGC-3’ |
| *TtNF-YA4A-EcoR*I | 5’-TTAGAATTCATGGCCGTGACGCTCTCCAC-3’ |
| *TtNF-YA4A-Xba*I | 5’-TTATCTAGACTAGGCGCCCCCTCTCGCGC-3’ |
| *TtNF-YA4A-1-EcoR*I | 5’-TTAGAATTCATGAGTGGCATGGGATCGCG-3’ |
| *TtNF-YA4A-1-Xba*I | 5’-TTATCTAGATCACGCCTGACGGAGATGCA-3’ |
| *TtNF-YA4B-1-EcoR*I | 5’-TTAGAATTCATGACTTCTGTCGCCGACGG-3’ |
| *TtNF-YA4B-1-Xba*I | 5’-TTATCTAGATCATTCATGGTTTCCCGACT-3’ |
| *TtNF-YA5A-2-EcoR*I | 5’-TTAGAATTCATGACTTCTGTCGCCGACGG-3’ |
| *TtNF-YA5A-2-Xba*I | 5’-TTATCTAGATCATTCATGGTTCCCCGACT-3’ |

**Table S2** List of *NF-YA* genes along with their gene ID used in this study.

| **Species** | **Gene Name** | **Gene ID** |
| --- | --- | --- |
| ***Triticum turgidum*** | *TtNF-YA2A-1* | TRITD2Av1G057050 |
|  | *TtNF-YA2B-1* | TRITD2Bv1G067630 |
|  | *TtNF-YA4A* | TRITD4Av1G003820 |
|  | *TtNF-YA4A-1* | TRITD4Av1G195760 |
|  | *TtNF-YA4B-1* | TRITD4Bv1G010020 |
|  | *TtNF-YA5A-1* | TRITD5Av1G012840 |
|  | *TtNF-YA5A-2* | TRITD5Av1G018890 |
|  | *TtNF-YA5A* | TRITD5Av1G198400 |
|  | *TtNF-YA5B-1* | TRITD5Bv1G010590 |
|  | *TtNF-YA5B-2* | TRITD5Bv1G019090 |
|  | *TtNF-YA6A-1* | TRITD6Av1G200860 |
|  | *TtNF-YA6B-1* | TRITD6Bv1G196010 |
| ***Arabidopsis thaliana*** | *AtNF-YA1* | AT5G12840:AT5G12840 |
|  | *AtNF-YA2* | AT3G05690:AT3G05690 |
|  | *AtNF-YA3* | AT1G72830:AT1G72830 |
|  | *AtNF-YA4* | AT2G34720:AT2G34720 |
|  | *AtNF-YA5* | AT1G54160:AT1G54160 |
|  | *AtNF-YA6* | AT3G14020:AT3G14020 |
|  | *AtNF-YA7* | AT1G30500:AT1G30500 |
|  | *AtNF-YA8* | AT1G17590:AT1G17590 |
|  | *AtNF-YA9* | AT3G20910:AT3G20910 |
|  | *AtNF-YA10* | AT5G06510:AT5G06510 |
| ***Oryza sativa*** | *OsNF-YA1* | Os08g0196700 |
|  | *OsNF-YA2* | Os12g0613000 |
|  | *OsNF-YA3* | Os03g0174900 |
|  | *OsNF-YA4* | Os03g0696300 |
|  | *OsNF-YA5* | Os03g0411100 |
|  | *OsNF-YA6* | Os03g0647600 |
|  | *OsNF-YA7* | Os07g0608200 |
|  | *OsNF-YA8* | Os03g0647600 |
|  | *OsNF-YA9* | Os10g0397900 |
|  | *OsNF-YA10* | Os07g0158500 |
| ***Triticum aestivum*** | *TaNF-YA1* | TraesCS6D02G315300 |
|  | *TaNF-YA2* | TraesCS5A02G373500 |
|  | *TaNF-YA3* | TraesCS4D02G289600 |
|  | *TaNF-YA4* | TraesCS2B02G198700 |
|  | *TaNF-YA5* | TraesCS5D02G052200 |
|  | *TaNF-YA6* | TraesCS5D02G383000 |
|  | *TaNF-YA7* | TraesCS4B02G290400 |
|  | *TaNF-YA8* | TraesCS2D02G179600 |
|  | *TaNF-YA9* | TraesCS2A02G172500 |
|  | *TaNF-YA10* | TraesCS6B02G366100 |
| ***Hordeum vulgare*** | *HvNF-YA1* | HORVU.MOREX. r3.2HG0126220 |
|  | *HvNF-YA2* | HORVU.MOREX. r3.4HG0404220 |
|  | *HvNF-YA3* | HORVU.MOREX. r3.6HG0619950 |
|  | *HvNF-YA4* | HORVU.MOREX. r3.5HG0508050 |
|  | *HvNF-YA5* | HORVU.MOREX. r3.4HG0335570 |
|  | *HvNF-YA6* | HORVU.MOREX. r3.5HG0426260 |
|  | *HvNF-YA7* | HORVU.MOREX. r3.5HG0429050 |
| ***Sorghum bicolor*** | *SbNF-YA1* | SORBI_3001G154500 |
|  | *SbNF-YA2* | SORBI_3001G340200 |
|  | *SbNF-YA3* | SORBI_3001G486000 |
|  | *SbNF-YA4* | SORBI_3002G038500 |
|  | *SbNF-YA5* | SORBI_3002G370800 |
|  | *SbNF-YA6* | SORBI_3008G174500 |
|  | *SbNF-YA7* | SORBI_3008G168300 |
|  | *SbNF-YA8* | SORBI_3008G174500 |
